# Innate Lymphoid Cells in Response to Intracellular Pathogens: Protection *Versus* Immunopathology

**DOI:** 10.3389/fcimb.2021.775554

**Published:** 2021-12-06

**Authors:** Anna A. Korchagina, Ekaterina Koroleva, Alexei V. Tumanov

**Affiliations:** Department of Microbiology, Immunology and Molecular Genetics, University of Texas Health Science Center at San Antonio, San Antonio, TX, United States

**Keywords:** innate lymphoid cells, intracellular pathogens, ILC plasticity, IFNγ, immunopathology

## Abstract

Innate lymphoid cells (ILCs) are a heterogeneous group of cytokine-producing lymphocytes which are predominantly located at mucosal barrier surfaces, such as skin, lungs, and gastrointestinal tract. ILCs contribute to tissue homeostasis, regulate microbiota-derived signals, and protect against mucosal pathogens. ILCs are classified into five major groups by their developmental origin and distinct cytokine production. A recently emerged intriguing feature of ILCs is their ability to alter their phenotype and function in response to changing local environmental cues such as pathogen invasion. Once the pathogen crosses host barriers, ILCs quickly activate cytokine production to limit the spread of the pathogen. However, the dysregulated ILC responses can lead to tissue inflammation and damage. Furthermore, the interplay between ILCs and other immune cell types shapes the outcome of the immune response. Recent studies highlighted the important role of ILCs for host defense against intracellular pathogens. Here, we review recent advances in understanding the mechanisms controlling protective and pathogenic ILC responses to intracellular pathogens. This knowledge can help develop new ILC-targeted strategies to control infectious diseases and immunopathology.

## Introduction

Numerous human infectious diseases are caused by intracellular pathogens that include bacteria, fungi, parasites, and viruses. In the majority of cases, infection is controlled by the host immune system which leads to the clearance of the pathogen in a relatively short period of time. However, infections with some intracellular pathogens result in long lasting course of disease accompanied by severe chronic inflammation with high morbidity and mortality, especially in individuals with compromised immune system (Thakur et al., 2019). Traditionally, intracellular pathogens are categorized into two groups based on their dependence on host cells: facultative and obligate intracellular pathogens (Leon-Sicairos et al., 2015; Casadevall and Fang, 2020). Facultative intracellular pathogens are capable of surviving and replicating both inside and outside the cell, whereas obligate intracellular pathogens entirely rely on intracellular resources of the host cell to reproduce and grow (Leon-Sicairos et al., 2015; Casadevall and Fang, 2020). Most pathogens invade the host through the barrier and mucosal surfaces such as skin, respiratory, reproductive, and digestive tracts. Pathogen invasion leads to acute inflammation, which is characterized by immediate and non-specific production of cytokines and chemokines, directing the innate lymphoid cells (ILCs) to the site of inflammation. Controlled acute immune response usually results in pathogen elimination and restoration of tissue homeostasis. However, dysregulated immune response that persists for a long time without resolution can lead to tissue damage and chronic inflammation.

**Figure 1:**
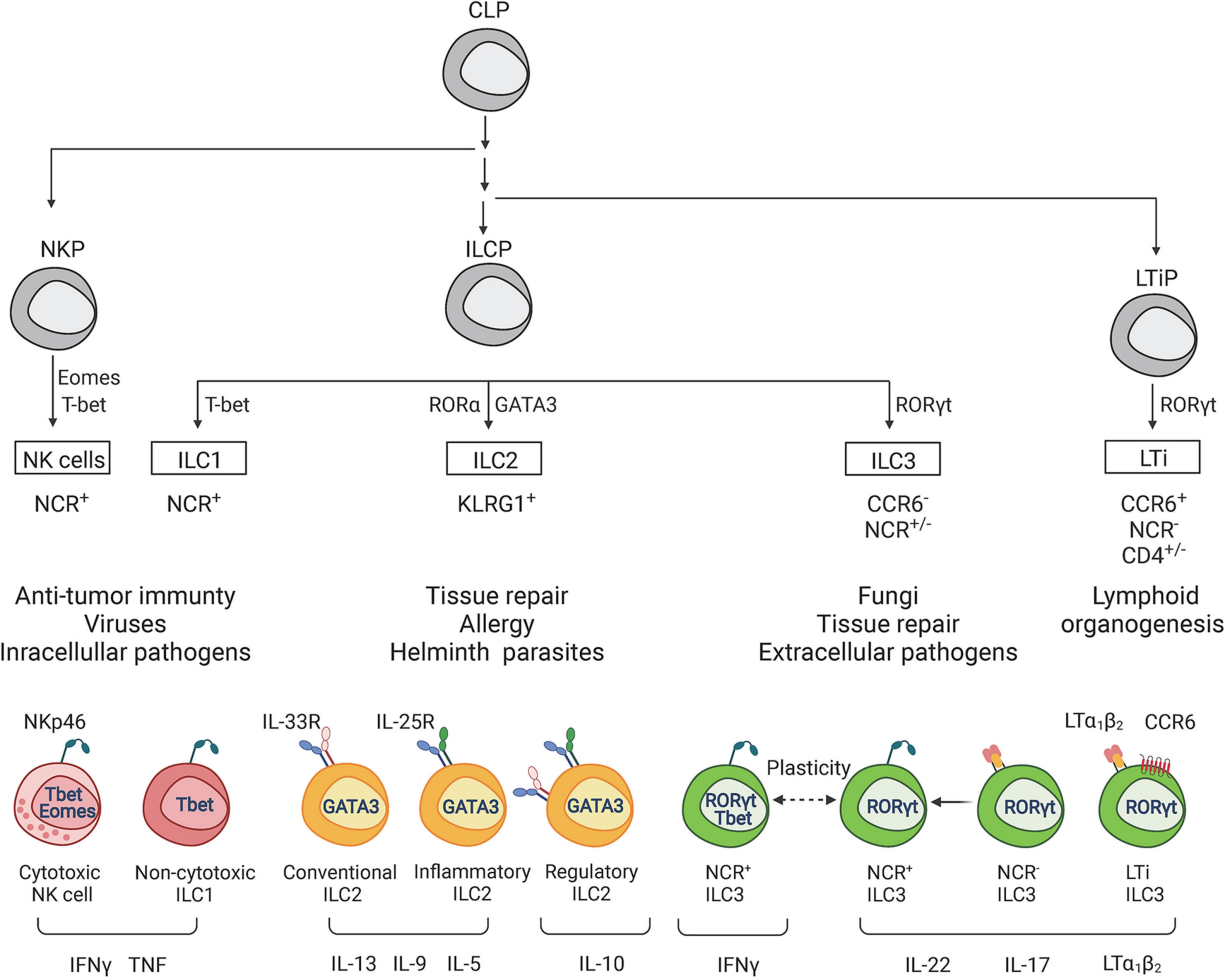
Classification of innate lymphoid cells. ILCs can be divided into 5 main subsets (NK cells, ILC1, ILC2, ILC3 and LTi cells) based on the differentiation stages, signature transcriptional factors and function. All ILCs develop from common lymphoid progenitor (CLP), which gives rise to three main subtypes of ILCs precursors: NK cell precursor (NKP), innate lymphoid cell precursor (ILCP), and lymphoid tissue inducer progenitor (LTiP). NKPs give rise to NK cells, ILCPs to ILC1s, ILC2s, and ILC3s, LTiPs – to LTi cells. Development and function of each ILC subset depends on corresponding specific transcriptional factors. Main signature transcriptional factors are illustrated: Eomes (Eomesodermin), T-bet (T-box transcriptional factor), GATA3 (GATA binding protein 3), RORγt (RAR-related orphan receptor γt). Additional transcriptional factors participate in subsequent cell lineage differentiation: ID2, PLZF, TCF7, TOX, NFIL3, RORα, AHR, RUNX3, BCL11b and others (not shown). IL-15 is required for the development of NK cells and ILC1s, whereas IL-7 and IL-2 are required for the development of ILC2s and ILC3s. NK cells and ILC1s contribute to defense against intracellular bacteria by producing TNF and IFNγ. NK cells are the cytotoxic ILCs which require Eomes for their development. Both ILC1s and NK cells express T-bet and produce IFNγ as a signature cytokine. ILC2 subsets contribute to immunity against large extracellular parasites and allergens by producing type 2 cytokines IL-13, IL-9, IL-10, IL-5. ILC2s are defined by high KLRG1 and GATA3 expression. Inflammatory and conventional ILC2s are classified based on response to IL-25 and IL-33 cytokines, respectively. ILC3s and LTis require RORγt expression for their development. ILC3s contribute to immune response against extracellular pathogens. ILC3 subsets include natural cytotoxicity receptor (NCR)^+^ ILC3s, NCR^-^ILC3s, and NCR^+^ILC1s derived from NCR^-^ILC3s due to plasticity (ex-ILC3s). IL-22, IL-17 and membrane lymphotoxin LTα_1_LTβ_2_ are key cytokines produced by ILC3s. In contrast to ILC3s, LTis express CCR6^+^ and CD4 but lack NCR. LTi contribute to the development of lymph nodes and gut-associated lymphoid tissues by expressing membrane lymphotoxin LTα_1_LTβ_2_ complex.

Interferon gamma (IFNγ) is known as one of the key cytokines which modulates the innate and adaptive immune responses against intracellular pathogens (Schroder et al., 2004; Kak et al., 2018). Although the majority of studies have been focused on the role of IFNγ-producing T cells in host protection against intracellular pathogens, recent studies revealed the crucial role of ILCs in orchestrating type I immunity to pathogens. ILCs are an emerging heterogeneous family of tissue-resident innate lymphocytes that are critical for maintaining mucosal tissue homeostasis and promoting early immune response during inflammation (Vivier et al., 2018). Mirroring the subsets of CD4^+^ effector T cells, ILCs are classified into five major groups: Natural killer cells (NK), group 1 ILCs (ILC1), group 2 ILCs (ILC2), group 3 ILCs (ILC3) and lymphoid tissue-inducer cells (LTi) (Vivier et al., 2018) (**Figure 1**). ILCs are enriched at mucosal surfaces, where they are located in close proximity to epithelial surfaces and rapidly initiate cytokine production in response to tissue damage and invading pathogens. The role of ILCs in regulation of protective responses to pathogens has been discussed in recent reviews (Beck et al., 2020; Seo et al., 2020). However, accumulating evidence suggests that dysregulated ILC responses can result in tissue damage and immunopathology (Buonocore et al., 2010; Vonarbourg et al., 2010; Bernink et al., 2013; Klose et al., 2013; Munoz et al., 2015; Muraoka et al., 2021). Additionally, dysregulation of ILCs can lead to chronic inflammation and autoimmunity, promoting diseases such as psoriasis (Villanova et al., 2014; Ward and Umetsu, 2014; Bielecki et al., 2021), atopic dermatitis (Brüggen et al., 2016), asthma (Cayrol and Girard, 2019) and inflammatory bowel disease (IBD) (Bernink et al., 2013; Bernink et al., 2015; Forkel and Mjosberg, 2016; Forkel et al., 2019). The mechanisms controlling the balance between protective and pathogenic responses mediated by ILCs remain poorly understood. In this review, we will focus on recent updates in ILC-dependent mechanisms that control protective and pathogenic responses induced by intracellular pathogens.

## ILC Subsets in Homeostatic Conditions and Disease

NK cells and ILC1s contribute to immune response against intracellular pathogens by producing TNF and IFNγ (Vivier et al., 2018; Seo et al., 2020). NK cells were initially included in the ILC1 group because of the similarities between NK cells and ILC1s. Thus, both populations express transcription factor T-bet and produce IFNγ in response to IL-12 stimulation. IL-15 is required for differentiation, homeostasis, and function of NK cells and ILC1s (Daussy et al., 2014). In addition to IFNγ, both NK cells and ILC1s can rapidly produce TNF in response to intracellular pathogens (Vivier et al., 2018; Seo et al., 2020). Similarly to NK cells, ILC1s express natural cytotoxicity receptors (NCRs) including NK1.1 and NKp46 (Fuchs et al., 2013). However, in contrast to NK cells, ILC1s require T-bet for their development, whereas NK cells need T-bet only for maturation but require Eomes for their differentiation (Gordon et al., 2012; Daussy et al., 2014). Moreover, NK cells and ILC1s arise from the different subpopulations of common lymphoid progenitors (CLPs): NK cells develop from NK cell precursors (NKP), whereas ILC1, 2, and 3 develop from innate lymphoid cell precursors (ILCP) (Klose et al., 2014; Vivier et al., 2018) (**Figure 1**). Therefore, based on the emerging functional, transcriptional, and epigenetic analyses of NK cells and ILC1s, they are now considered as distinct lineages within the ILC family.

ILC2s are essential for control of helminth infections (Neill et al., 2010; Price et al., 2010) and tissue repair (Palm et al., 2012) and are involved in pathogenesis of asthma and allergy (Ho et al., 2015; Silver et al., 2016; Xiao et al., 2021) by secretion of type 2 cytokines IL-5, IL-9 and IL-13. Moreover, IL-2 and IL-7 are required for both ILC2s and ILC3s development and function (Vivier et al., 2018). ILC2s require the transcription factors GATA3 and RORα for their development and maintenance (Mjosberg et al., 2012; Wong et al., 2012; Walker and McKenzie, 2013; Ferreira et al., 2021). ILC2s are classified into two subtypes based on their response to distinct cytokines. Inflammatory ILC2 (iILC2s) respond to IL-25 and contribute to helminth expulsion, whereas natural ILC2s (nILC2s) are mainly present at a steady state and respond to IL-33 (Huang et al., 2015; Huang and Paul, 2016). Recently, a new population of ILC2s was identified in the lungs, which can produce IL-10 in response to IL-33 (Seehus et al., 2017; Golebski et al., 2021; Howard et al., 2021). IL-10-producing ILC2s are characterized by decreased expression of proinflammatory genes and have reduced ability to recruit eosinophils to the lungs (Seehus et al., 2017). Moreover, these cells have a distinct transcriptional profile which separates them from recently identified IL-10 producing ILCs in the gut named ILCregs (Seehus et al., 2017). Recent studies showed that ILC2s can upregulate the expression of T-bet and acquire ILC1-like effector program in inflamed lung tissue of patients with chronic obstructive pulmonary disease (COPD) as well as in inflamed nasal tissue (Bal et al., 2016; Silver et al., 2016). However, IL-10-producing ILC2s fail to acquire T-bet and IFNγ expression upon IL-33-mediated activation, suggesting that they are distinct from IFNγ-producing ILC1-like cells (Seehus et al., 2017). Additionally, a recent study demonstrated that human ILC2s produce IL-10 to maintain epithelial integrity upon allergen exposure and can inhibit Th2 responses in an IL-10-dependent manner (Golebski et al., 2021). Although ILC2s have been extensively studied in the lungs, limited data is available on the role of ILC2s in the intestine. So far, the protective role of ILC2s activated by IL-33 was demonstrated in DSS colitis model (You et al., 2020; Ngo Thi Phuong et al., 2021) in which activated ILC2s produced IL-13 thereby inducing goblet cell expansion and intestinal barrier repair (Klose et al., 2017). Additionally, IL-13 from ILC2s may promote M2 macrophage polarization leading to reduced intestinal inflammation (You et al., 2020). However, the role of ILC2s in intestinal inflammation in humans remains largely unexplored. An increased ILC2 numbers have been reported in IBD patients (Lim et al., 2016; Forkel et al., 2019). Recent study revealed an increase of SLAMF1^+^ (Signaling Lymphocytic Activation Molecule Family Member 1) ILC2s in the intestine of Crohn’s disease patients compared to healthy individuals (Mazzurana et al., 2021). Moreover, the frequency of SLAMF1^+^ ILC2s negatively correlated with disease progression (Mazzurana et al., 2021). Another study found that SLAMF1 expression was associated with IL-13 expression by ILC2s in the lung (Mazzurana et al., 2021). However, IL-13-producing ILC2s from intestinal samples of Crohn’s disease patients can also produce IFNγ (Lim et al., 2016), which could contribute to intestinal pathology. Thus, the role of ILC2s in intestinal inflammation needs to be further clarified.

Several studies showed that ILC2s activation can be controlled by neuroregulators such as neuromedin U (NMU) (Nagashima et al., 2019; Pu et al., 2021). ILC2s are located in close proximity to cholinergic neurons that secrete NMU in the intestine and lungs (Cardoso et al., 2017; Klose et al., 2017; Wallrapp et al., 2017). ILC2s express high levels of NMU receptor, Nmur1, which is required to induce type 2 cytokines for protection against worm infection in the lungs and intestine (Cardoso et al., 2017; Wallrapp et al., 2017). Interestingly, NMU triggers immediate production of IL-5, IL-13 and IL-9 by ILC2s, whereas IL-33 and IL-25 stimulation delays activation of ILC2s, suggesting rapid neuronal-dependent activation of immune mechanisms in response to changing environmental cues (Cardoso et al., 2017; Klose et al., 2017). Additionally, ILC2s activated by NMU produce IL-9, which in turn induces expansion of IL-17A-producing γδ T cells in the lung during sepsis (Chen et al., 2021). NMU regulates ILC2 protective responses through induction of IL-10, which inhibits IL-33-mediated eosinophil recruitment to the lungs (Bando et al., 2020). Another neuropeptide, Calcitonin gene-related peptide (CGRP), suppresses IL-33- and NMU-activated ILC2s proliferation and IL-13 production but promotes IL-5 secretion during helminth infection (Vivier et al., 2009; Nagashima et al., 2019). These studies demonstrate the complexity of neuropeptide- ILC2s interactions, which are context- and tissue-dependent.

Recent findings indicate that ILC2s can migrate from the intestine to the lungs in response to helminth infection or cytokine stimulation (Huang et al., 2018; Pu et al., 2021). Moreover, gut microbiome can influence the immune responses locally or systemically. Stomach microbiota induces IL-7 and IL-33 production, leading to expansion of ILC2s. In turn, ILC2s produce IL-5 for IgA-mediated control of *Helicobacter pylori* in the stomach (Satoh-Takayama et al., 2020). Gut microbiota can also contribute to asthma development in infants *via* regulation of ILC2s effector function (Chua et al., 2018). Intestinal dysbiosis activates colon epithelial cells to secrete IL-33, IL-25 and TSLP, leading to IL-13 production by ILC2s to induce migration of dendritic cells and differentiation of Th2 cells (Chua et al., 2018). IL-5 produced by ILC2s and Th2 cells recruits eosinophils to colon and lungs, thereby promoting asthma development (Chua et al., 2018). While the detailed mechanism of microbiota-mediated regulation of ILC2s migration from the gut to the lungs remains to be determined, it has been recently demonstrated that IL-33/CXCL16 axis regulates migration of nILC2 to the lungs, whereas IL-25/CCL25 axis facilitates migration of iILC2s to the intestine during infection (Pu et al., 2021).

ILC3s predominantly respond to extracellular pathogens and fungi by producing type 3 cytokines (IL-22, IL-17) and lymphotoxin (LT) (Colonna, 2009; Vivier et al., 2009; Tumanov et al., 2011; Kruglov et al., 2013). The development and differentiation of ILC3s is dependent on the constitutive expression of transcription factor retinoic acid-related orphan receptor γt (RORγt). Accordingly, RORγt-deficient mice lack ILC3s (Cupedo et al., 2009; Sanos et al., 2009; Buonocore et al., 2010). ILC3s express the membrane complex LTα_1_LTβ_2_ and soluble LTα_3_, which signal to lymphotoxin beta receptor (LTβR) and TNFRs respectively (Tumanov et al., 2011; Kruglov et al., 2013). LTα_1_LTβ_2_ is not required for the development of ILC3s and LTi cells but contributes to IL-22 production by these cells *via* ILC3s interactions with dendritic cells and intestinal epithelial cells (Tumanov et al., 2011; Macho-Fernandez et al., 2015). Accordingly, mice lacking LTβ on ILC3s fail to produce IL-22 in response to the mucosal bacterial pathogen *Citrobacter rodentium* (*C. rodentium*) and succumb to infection (Wang et al., 2010; Tumanov et al., 2011). LT expression by ILC3s also controls the development of NK cells *via* LTβR signaling on stromal cells (Kim et al., 2014; Shou et al., 2021). Additionally, aryl hydrocarbon receptor (Ahr) controls the maintenance and postnatal expansion of ILC3s (Kiss and Diefenbach, 2012; Klose et al., 2013). ILC3s are divided into at least two subsets based on expression of natural cytotoxicity receptor (NCR): NCR^+^ILC3s and NCR^-^ILC3s. NCR^+^ILC3s require T-bet for their differentiation from NCR^-^ILC3 precursor cells (Cupedo et al., 2009; Sanos et al., 2009; Klose et al., 2013). Moreover, ILC3s can undergo conversion toward ILC1s (Klose et al., 2013; Bernink et al., 2015). The plasticity between ILC populations is controlled by distinct cytokines and transcription factors (Bal et al., 2020). For example, NCR^-^ILC3 to NCR^+^ILC1 plasticity is controlled by the balance of RORγt and T-bet (Vonarbourg et al., 2010; Bal et al., 2020).

LTi cells are critical for the development of lymph nodes and Peyer’s patches (PP) during embryogenesis (Mebius et al., 1997; Eberl et al., 2004). Expression of surface LTα_1_LTβ_2_ complex on LTi cells is critical for the development of lymph nodes, PPs, isolated lymphoid follicles, and cryptopatches in the intestine *via* interactions with LTβR on lymphoid tissue organizer cells (Bar-Ephraïm and Mebius, 2016; Onder and Ludewig, 2018). Accordingly, mice lacking surface lymphotoxin on LTi cells fail to develop lymph nodes and gut-associated lymphoid tissues (Kruglov et al., 2013). Fetal LTi cells are replaced by bone-marrow derived hematopoietic stem cells during adulthood (Simic et al., 2020; van de Pavert, 2021). However, the role of LTi cells in mucosal immune response in adulthood remains poorly understood. CCR6^+^LTi-like cells are the adult counterpart of LTi cells, which participate in formation of isolated lymphoid follicles and cryptopatches *via* LTα_1_LTβ_2_ expression and remodeling of secondary lymphoid organs during infection and inflammation (Scandella et al., 2008; Colonna, 2009; Vivier et al., 2009). Additionally, ILC3s can regulate adaptive immune response directly by cell contact-dependent interactions or indirectly *via* cytokine production (Hepworth et al., 2013; Hepworth et al., 2015; Castellanos et al., 2018; Rao et al., 2020).

ILC3s play a critical role in maintaining epithelial cells integrity during homeostasis and regulate rapid repair of epithelial barrier during inflammation (Vivier et al., 2018). ILC3s can be activated by multiple signals including cytokines, metabolites, microbial and neuronal signals (Chun et al., 2019). Myeloid-derived IL-23 and IL-1β activate ILC3s to produce IL-22 that targets epithelial and stromal cells to induce proliferation and release of antimicrobial peptides in response to *C. rodentium* (Tumanov et al., 2011; Qiu et al., 2012; Melo-Gonzalez and Hepworth, 2017). Glial cell-derived neurotrophic factor family ligands through neuroregulatory receptor RET promote IL-22 secretion by ILC3s (Ibiza et al., 2016). ILC3s also secrete IL-22 in response to vasoactive intestinal peptide (VIP) produced by enteric neurons *via* VIPR2 (Seillet et al., 2020; Talbot et al., 2020). Recent studies revealed additional pathways which contribute to IL-22 production by ILC3s. Epithelial cell-derived IL-17D can induce IL-22 production by ILC3s through CD93 receptor during physiological and pathological conditions (Huang et al., 2021). Additionally, in response to bacterial metabolites such as short-chain fatty acids, free fatty acid receptor 2 (Ffar2) on colonic ILC3s contributes to IL-22 production (Chun et al., 2019). Besides the essential role of ILC3s in tissue repair, ILC3s can directly recognize neutrophil apoptosis during intestinal inflammation and after skin damage (Wang et al., 2021). Neutrophils, which undergo apoptosis during intestinal inflammation, release lysophosphatidylserine which can be sensed by ILC3s through GPR34 resulting in IL-22 production (Wang et al., 2021). Accordingly, mice lacking CD93, GPR34, or Ffar2 expression on ILC3s showed reduced IL-22 levels in the colon and increased bacterial burden following *C. rodentium* infection, although mice were able to survive the infection (Chun et al., 2019; Huang et al., 2021; Wang et al., 2021). Thus, multiple redundant pathways regulate IL-22 production by ILC3s to ensure robust IL-22 response to protect against mucosal pathogens and tissue damage.

Dynamic changes caused by chronic inflammation can result in phenotypical and functional changes in ILCs composition thereby contributing to disease pathology in patients with IBD (Geremia et al., 2011; Bernink et al., 2013; Bernink et al., 2015; Forkel et al., 2019). For example, number of ILC1s is increased whereas NCR^+^ILC3 subset is reduced in inflamed tissue of IBD patients (Takayama et al., 2010; Bernink et al., 2013; Forkel et al., 2019) and changes in ILCs composition correlated with disease severity (Forkel et al., 2019). Physiologically, ILC2s represent the major ILC subset in the skin, however during chronic inflammatory skin disease ILC2s undergo transition to ILC3s (Bielecki et al., 2021). Moreover, psoriasis patients treated with TNF blockers display reduced numbers of ILC3s suggesting their role in psoriasis pathogenesis (Villanova et al., 2014). In contrast to psoriasis, ILC2s are increased in patients with atopic dermatitis and ILC2s promote atopic dermatitis-like lesions in mouse models (Kim et al., 2013; Salimi et al., 2013; Imai et al., 2019). Thus, these studies suggest that the plasticity or disbalance between ILC subsets can contribute to pathogenesis of inflammatory diseases.

## Protective and Pathogenic Effects of IFNγ Produced by ILCs in Response to Intracellular Pathogens

**Figure 2:**
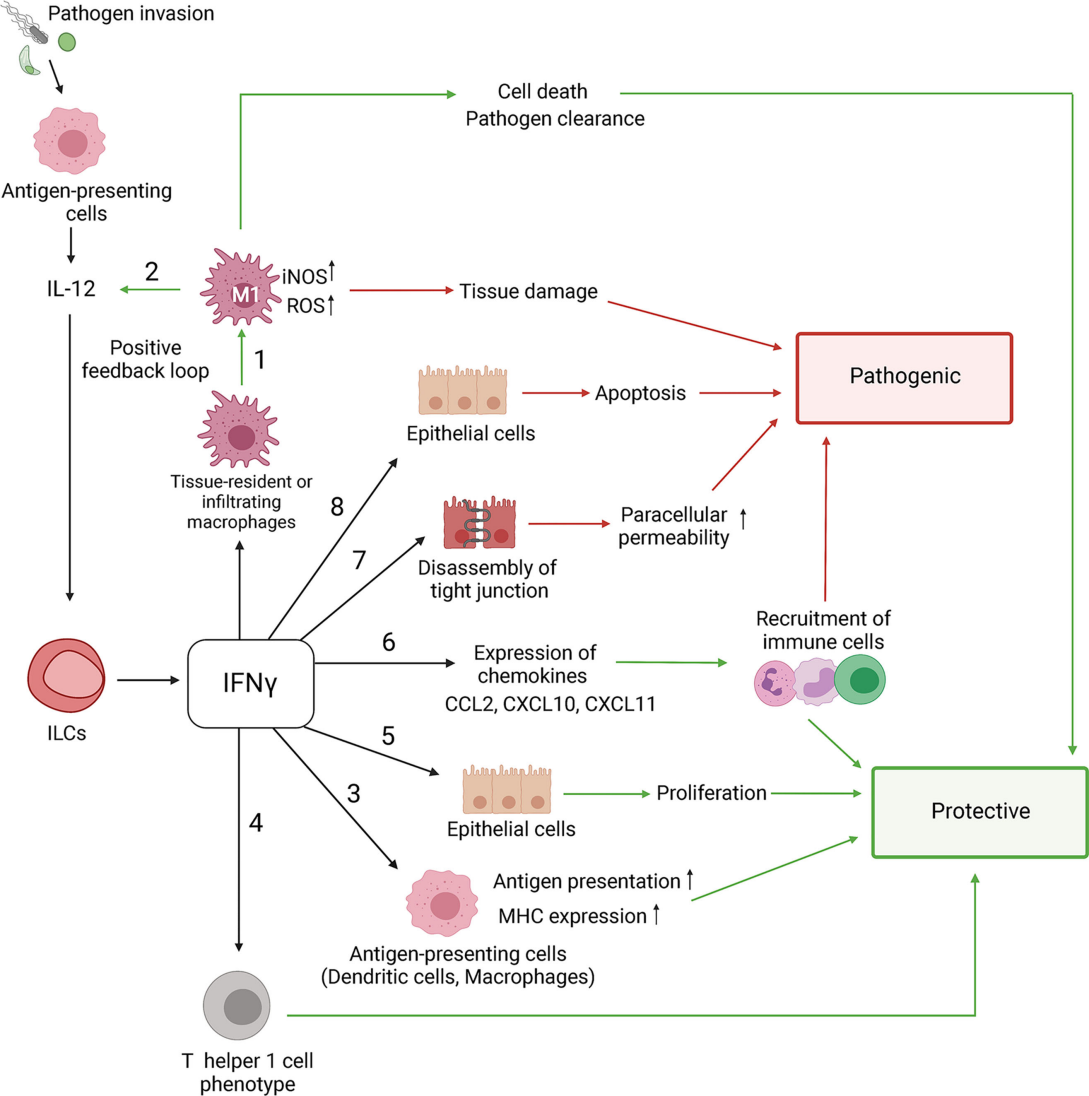
Role of IFNγ in protective and pathogenic responses to intracellular pathogens. The IFNγ-mediated effects are context-dependent and can contribute to both pathogen clearance and tissue damage. NK cells, ILCs and T cells are primary producers of IFNγ under inflammatory conditions. 1) IFNγ induces polarization of M1 macrophages that produce high levels of reactive oxygen species (ROS) and nitric oxide (NO) which results in death of pathogen-infected cells and tissue damage. 2) IFNγ stimulates antigen-presenting cells to produce IL-12, which in turn induces more IFNγ production by ILCs, NK and T cells through a positive feedback loop. 3) IFNγ induces MHC I and MHC II expression which promotes antigen presentation. IFNγ stimulates: 4) Th1 T cell polarization, 5) proliferation of epithelial cells and 6) expression of inducible CXCL9, CXCL10, CXCL11 chemokines that recruit various immune cells *via* CXCR3. However, sustained activation of IFNγ-R signaling can lead to chronic inflammation and tissue damage. 7) IFNγ regulates epithelial barrier permeability by altering intercellular tight junctions leading to increased permeability to intestinal bacteria and pathogens. 8) IFNγ can exacerbate intestinal inflammation through inhibition of epithelial cell proliferation and promotion of apoptosis. Green/red arrows indicate protective/pathogenic responses.

IFNγ can be synthesized by various immune cells such as NK cells and antigen-specific T cells (Okamura et al., 1998; Frucht et al., 2001). However, recent studies demonstrated that ILC1s and ILC3s can also secrete IFNγ in response to activation (Vivier et al., 2018). Accumulating evidence suggests that IFNγ produced by ILCs contributes to early host defense against pathogens, whereas IFNγ produced by T cells is critical for adaptive immune response. IFNγ secreted at early stages of infection enhances antigen presentation by antigen-presenting cells (APCs), such as macrophages and dendritic cells, by inducing MHC expression (Amaldi et al., 1989; Steimle et al., 1994; Xaus et al., 2000; Schroder et al., 2004). Additionally, APCs secrete IL-12, IL-15, and IL-18, which stimulate IFNγ secretion at the infection site. Moreover, IL-12 can synergize with IL-18 to enhance IFNγ production (Okamura et al., 1998; Tominaga et al., 2000; Frucht et al., 2001; Tait Wojno et al., 2019). IFNγ production by ILCs is strictly regulated by lineage-defining and signal-dependent transcriptional factors and cytokines (Mikami et al., 2018; Stabile et al., 2018). IL-12 binding to IL-12 receptor leads to STAT4 phosphorylation and induction of IFNγ expression. Moreover, IFNγ production by ILC1s and NCR^+^ILC3s is amplified by high levels of STAT4 expression induced by T-bet (Mikami et al., 2018; Yin et al., 2018). Additionally, coordinated expression of STAT4 and T-bet induces IFNγ production by ILC3s after IL-23 activation (Mikami et al., 2018). Furthermore, Runx3 transcriptional factor modulates IL-12/STAT4 axis to promote secretion of IFNγ by ILCs during bacterial infection (Yin et al., 2018). Bacterial lipopolysaccharides and other pathogen-associated molecular patterns directly induce IL-12 production by activated monocytes, macrophages, neutrophils and dendritic cells. In turn, IL-12 can further amplify IFNγ by ILCs and T cells (Darwich et al., 2009; Tait Wojno et al., 2019) (**Figure 2**). Although both human and mouse ILCs express TLRs (Crellin et al., 2010; Maggi et al., 2017), it has yet to be demonstrated if activation of TLRs can directly induce IFNγ production by ILCs. IFNγ stimulates expression of CXCL9, CXCL10, and CXCL11 chemokines which promote CXCR3-dependent recruitment of T cells and ILCs (Metzemaekers et al., 2017). In addition, IFNγ activates macrophages, leading to increased phagocytosis as well as enhanced production of reactive nitric oxide (NO), and indoleamine 2,3-dioxygenase (IDO)-mediated tryptophan depletion (Decker et al., 2002; Popov et al., 2006) (**Figure 2**). NO is generated in immune cells from L-arginine and oxygen *via* inducible NO synthase (iNOS) (Bogdan, 2015). iNOS contributes to protection against intracellular pathogens such as *Toxoplasma gondii* (Dunay and Diefenbach, 2018), *Listeria monocytogenes* (Lu et al., 2015), *Mycobacterium tuberculosis* (Pahari et al., 2016; Braverman and Stanley, 2017) and *Salmonella typhimurium* (Yadav et al., 2020), as mice with inactivation of *Nos2* gene are highly susceptible to these infections. The induction of IDO1 and consequent tryptophan degradation is an effective antimicrobial mechanism accelerated in response to IFNγ. For instance, IDO1 activation is an effective inhibitor of *T. gondii* replication (Dunay and Diefenbach, 2018), while IDO was shown to be not essential for control of *M. tuberculosis* (Blumenthal et al., 2012).

In contrast to protective effects of IFNγ in response to intracellular pathogens, an increased or prolonged IFNγ production results in tissue damage. For example, during the onset of inflammation, IFNγ promotes epithelial cell proliferation through activation of AKT-β-catenin signaling pathway, which is potentiated by TNF (Nava et al., 2010). However, extended IFNγ and AKT-β-catenin activation inhibits epithelial cell proliferation and induces apoptosis by activating Wnt inhibitor DKK1 (Nava et al., 2010). Increased IFNγ production can also induce depletion of Paneth cells which produce cytotoxic antibacterial proteins and promote proliferation of Lgr5^+^ intestinal stem cells (Eriguchi et al., 2018).

Epithelial barrier integrity is critical to restrict entrance of pathogens to the host and maintain tissue homeostasis. Intercellular tight junctions are critical for the maintenance of epithelial homeostasis (Chelakkot et al., 2018). Altered tight junction complexes result in increased epithelial cell permeability to pathogens and commensal bacteria, leading to altered barrier functions, production of inflammatory cytokines, and tissue damage (May et al., 1993; Zeissig et al., 2007; van der Gracht et al., 2016). Activation of IFNγ signaling in epithelial cells can result in the weakening of intercellular tight junctions, thereby promoting permeability of the epithelial barrier. Indeed, biopsy samples from IBD patients demonstrated disrupted tight junctions and increased epithelial cell permeability (Schmitz et al., 1999; Zeissig et al., 2007; Ahmad et al., 2017; Odenwald and Turner, 2017). In addition to impaired tight junctions, high rates of apoptosis in epithelial cells are also linked to IBD development (Zeissig et al., 2007; Chelakkot et al., 2018). Collectively, IFNγ orchestrates numerous protective pathways to maintain tissue homeostasis and induce rapid immune response to reduce microbial burdens. However, excessive production of IFNγ can lead to exacerbation of immune response, tissue damage, and chronic inflammation (**Figure 2**).

## ILCs in Immune Response to Intracellular Pathogens

### Toxoplasma gondii

*Toxoplasma gondii (T. gondii*) is an obligate protozoan parasite that can infect all warm-blooded vertebrates and cause toxoplasmosis (Konradt et al., 2016; Dunay and Diefenbach, 2018). Infection with *T. gondii* may lead to parasite dissemination to the central nervous system and muscle tissue where *T. gondii* converts to bradyzoite-containing cysts that remain lifelong in the host (Konradt et al., 2016; Dunay and Diefenbach, 2018).

**Figure 3:**
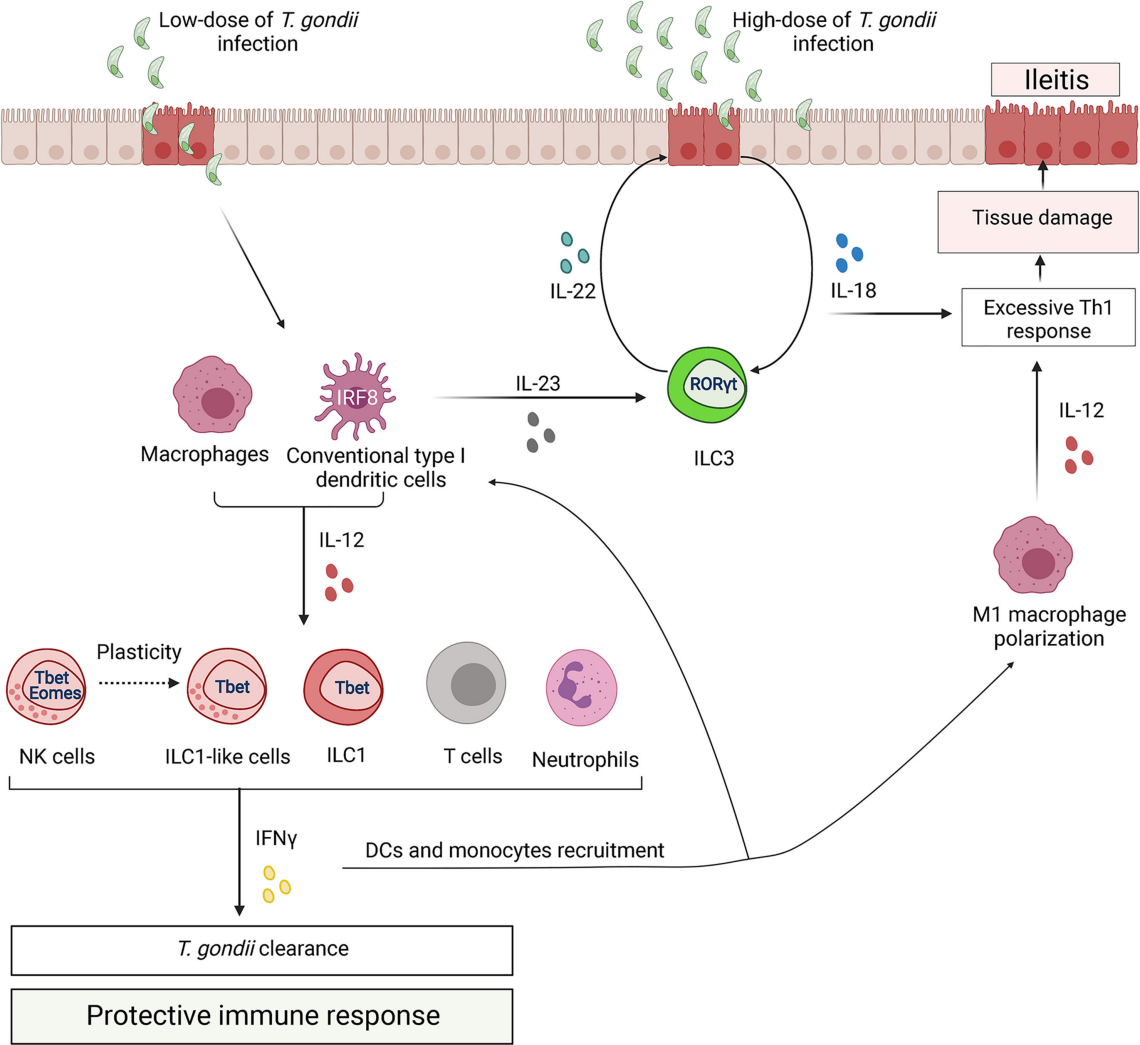
ILCs regulate the immune response to *Toxoplasma gondii*. Control of parasite is associated with type 1 immune response. Upon infection, parasite infects ileum enterocytes. In response to *T. gondii* invasion, epithelial cells release cytokines (IL-8, CCL5 and CCL3), which activate antigen-presenting cells, particularly dendritic cells (DCs) and macrophages (MФ). Additionally, *T. gondii* can directly activate DCs and MФ through TLR11 and TLR12. Parasite recognition by DCs leads to production of IL-12, which primes NK cells, ILCs and T cells to secrete IFNγ. IFNγ production leads to parasite clearance. Additionally, IFNγ from ILC1s induces transcriptional factor IRF8 for differentiation of type I conventional DCs. *T. gondii* infection drives plasticity of NK cells to ILC1-like cells by downregulating expression of Eomes. In contrast, pathogenic IFNγ promotes polarization of M1 macrophages. M1 macrophages produce more IL-12 leading to excessive activation of CD4^+^ T cells that can lead to tissue damage. Moreover, upon *T. gondii* infection intestinal epithelial cells produce IL-18 that enhances IFNγ induction. Additionally, DCs-derived IL-23 synergizes with IL-18 to induce IL-22 production by ILC3s. ILC3-driven IL-22 enhances production of IL-18 by epithelial cells resulting in amplification of pathogenic positive feedback loop that exacerbates Th1-induced immune response.

IFNγ and IL-12 production is critical for protection against *T. gondii*, as IFNγ^-/-^ mice or IL12p35^-/-^ mice succumb to infection (Suzuki et al., 1988; Scharton-Kersten et al., 1996; Lieberman et al., 2004). Activation of DCs, macrophages, and neutrophils in response to *T. gondii* results in the production of proinflammatory cytokines, including IL-12, which primes NK cells, ILCs, and T cells to secrete IFNγ (Gazzinelli et al., 1994; Mashayekhi et al., 2011) (**Figure 3**). While NK cells are important for host resistance at early onset of *T. gondii*-mediated disease, adaptive immunity mainly contributes to protection during chronic phase of infection (Denkers et al., 1993). NK cells produce IFNγ which induces parasite destruction (Denkers et al., 1993; Yarovinsky, 2014). Insufficient IFNγ production by NK cells during *T. gondii* infection is associated with reduced numbers of IFNγ-producing CD4^+^ and CD8^+^ T cells (Goldszmid et al., 2007; Ivanova et al., 2020). Additionally, IFNγ produced by NK cells promotes differentiation of monocytes into inflammatory IL-12-producing DCs which contribute to protection (Goldszmid et al., 2012). Recent studies indicate that NK cells not only contribute to early protection against *T. gondii* but can also inhibit CD8^+^ T cells in chronic toxoplasmosis (Ivanova et al., 2020). Thus, it was shown that during chronic *T. gondii* infection, NK cells had reduced IFNγ production and increased expression of CD107a – a surface marker of NK cell activity (Ivanova et al., 2020). Depletion of these NK cells with anti-NK1.1 antibody rescued chronic *T. gondii* infected mice from CD8^+^ T cell exhaustion-dependent death (Ivanova et al., 2020). NK cell depletion reduced CD8^+^ T cell apoptosis, indicating that NK cells can contribute to CD8^+^ T cell exhaustion by promoting apoptosis (Ivanova et al., 2020). However, the molecular mechanisms by which NK cells inhibit CD8^+^ T cells remain unclear.

Recent studies demonstrated that along with NK cells, ILC1s also contribute to host defense against *T. gondii* and to parasite clearance (Klose et al., 2014; Lopez-Yglesias et al., 2021). Genetic deletion of T-bet resulted in profound loss of IFNγ production due to a deficiency of ILC1s, despite unaffected NK cell numbers in the small intestine during *T. gondii* infection (Klose et al., 2014). Furthermore, these studies suggest that ILC1s are a primary cellular source of IFNγ in the small intestine rather than NK cells or NKp46^+^NK1.1^+^ILC3s.

Inflammatory monocytes directly limit early *T. gondii* replication, whereas DCs are the major cellular source of IL-12, which is produced upon activation of pattern recognition receptors by parasite (Dunay et al., 2008). More recently, it became evident that ILC1s and NK cells promote the recruitment of inflammatory monocytes and DCs to protect against *T. gondii* (Goldszmid et al., 2012; Klose et al., 2014). Thus, T-bet-deficient mice lacking IFNγ-producing ILC1s showed significant reduction of IRF8^+^ inflammatory DCs and succumbed to *T. gondii* (Lopez-Yglesias et al., 2021). Moreover, infection with *T. gondii* promotes stromal cells to produce IL-33, which synergizes with IL-12 to amplify IFNγ production by ILCs for protection (Clark et al., 2021). These data suggest that IL-12, IL-33 and IFNγ mediate the crosstalk between ILC1s, stromal cells and DCs to protect against *T. gondii* infection.

*T. gondii* infection results in immunopathology associated with Th1 immune response (Yarovinsky, 2014; Dunay and Diefenbach, 2018) (**Figure 3**). It is important to note that the parasite dose, strain, and infection route can influence the outcome of infection (Dunay and Diefenbach, 2018). Thus, infections with low parasite cyst numbers (<20 cysts) or with less virulent strains lead to protective Th1 immune response, whereas oral infections with higher parasite doses cause extensive production of proinflammatory cytokines, leading to exacerbation of ileitis induced by *T. gondii* (Munoz et al., 2011). These differences may explain contradictory reports on the role of IL-22 and IL-18 in *T. gondii*-mediated intestinal pathology (Munoz et al., 2009; Wilson et al., 2010; Munoz et al., 2015; Couturier-Maillard et al., 2018). Thus, sustained IL-22 and IL-18 production promoted intestinal inflammation in the high dose oral infection model, as IL-22^-/-^ and IL-18^-/-^ mice exhibited significantly less infection-induced ileitis (Munoz et al., 2015). However, there was no difference in tissue pathology and the number of IFNγ^+^ cells between WT and IL-22^-/-^ mice following low-dose of intraperitoneal infection with *T. gondii* (Wilson et al., 2010). IL-18 is known to protect against intracellular pathogens by amplifying IFNγ production together with IL-12 (Frucht et al., 2001; Shtrichman and Samuel, 2001; Ivashkiv, 2018). However, excessive production of IL-18 by epithelial cells after infection with high dose of *T. gondii* contributes to intestinal pathology by inducing IFNγ (Munoz et al., 2015). In contrast, IL-18 only slightly induces IFNγ after low dose of infection (Vossenkamper et al., 2004; Munoz et al., 2009; Dunay and Diefenbach, 2018). Furthermore, after high dose of oral *T. gondii* infection, IL-18 synergizes with IL-23 for production of IL-22 (Munoz et al., 2015). DC-derived IL-23 induces IL-22 production by ILC3s. In turn, IL-22 induces IL-18 production by epithelial cells, thereby amplifying this pathogenic feedback loop (Wilson et al., 2010; Munoz et al., 2015; Victor et al., 2017) (**Figure 3** **)**. Whether IL-22 can contribute to *T. gondii*- induced intestinal pathology independently from IL-18 remains unclear.

Recently the first study describing infection-induced conversion of NK cells to ILC1-like cells was published in *T. gondii* infection model (Park et al., 2019). A previous study described the conversion of NK cells to ILC1-like cells within the tumor microenvironment (Gao et al., 2017), but, in contrast to tumor ILC1-like cells, *T. gondii*-induced ILC1-like cells were not tissue-resident but were able to circulate under inflammatory conditions (Park et al., 2019). Interestingly, ILC1-like cells were maintained after the infection was cleared, similarly to immune memory NK cells (Park et al., 2019). These findings suggest a previously underappreciated plasticity between NK cells and ILCs in immune response to *T. gondii* infection. The physiological significance of NK cell conversion to ILC1-like cells and the mechanisms that regulate this plasticity remain unknown and require further studies.

Although NK cells and ILC1s are required for IFNγ-dependent resistance against *T. gondii* in mouse models, the role of ILCs in human *T. gondii* infection and how findings in mice translate to human diseases remains to be determined. Mice as an intermediate *T. gondii* host potentially developed unique resistance mechanism which could be different from response in humans. Humanized animal models could provide a useful tool to address the role of human ILCs in response to parasite. Recent studies revealed that NK cells and ILC1s are present in the central nervous system (CNS) of mice (Romero-Suárez et al., 2019). Moreover, it has been shown that ILC1s can be recruited to CNS during experimental autoimmune encephalomyelitis and control the onset of neuroinflammation (Kwong et al., 2017). Since previous studies of ILCs were mostly focused on the periphery rather than CNS, it will be important to examine the role of ILCs in cerebral toxoplasmosis.

### Salmonella typhimurium

*Salmonella* is a facultative intracellular bacterium which causes food-borne infectious gastroenteritis in humans called salmonellosis. The clinical disease in humans and animals is mainly caused by non-typhoidal *Salmonella typhimurium* and *S. enteritidis*, and usually is self-limiting. However, the clinical outcome of infection depends on the immunological status of the individual and serovar of bacteria. *Salmonella* infection can cause systemic disease that can be fatal, especially for immunocompromised individuals (Pham and McSorley, 2015). Although microfold cell (M cells) and DCs in PPs are the main entry points for *Salmonella*, the bacterium can also use other routes for invasion, such as intestinal epithelium, and can disseminate and replicate in the spleen, liver, and phagocytic cells in bone marrow (Santos and Bäumler, 2004; Tahoun et al., 2012).

**Figure 4:**
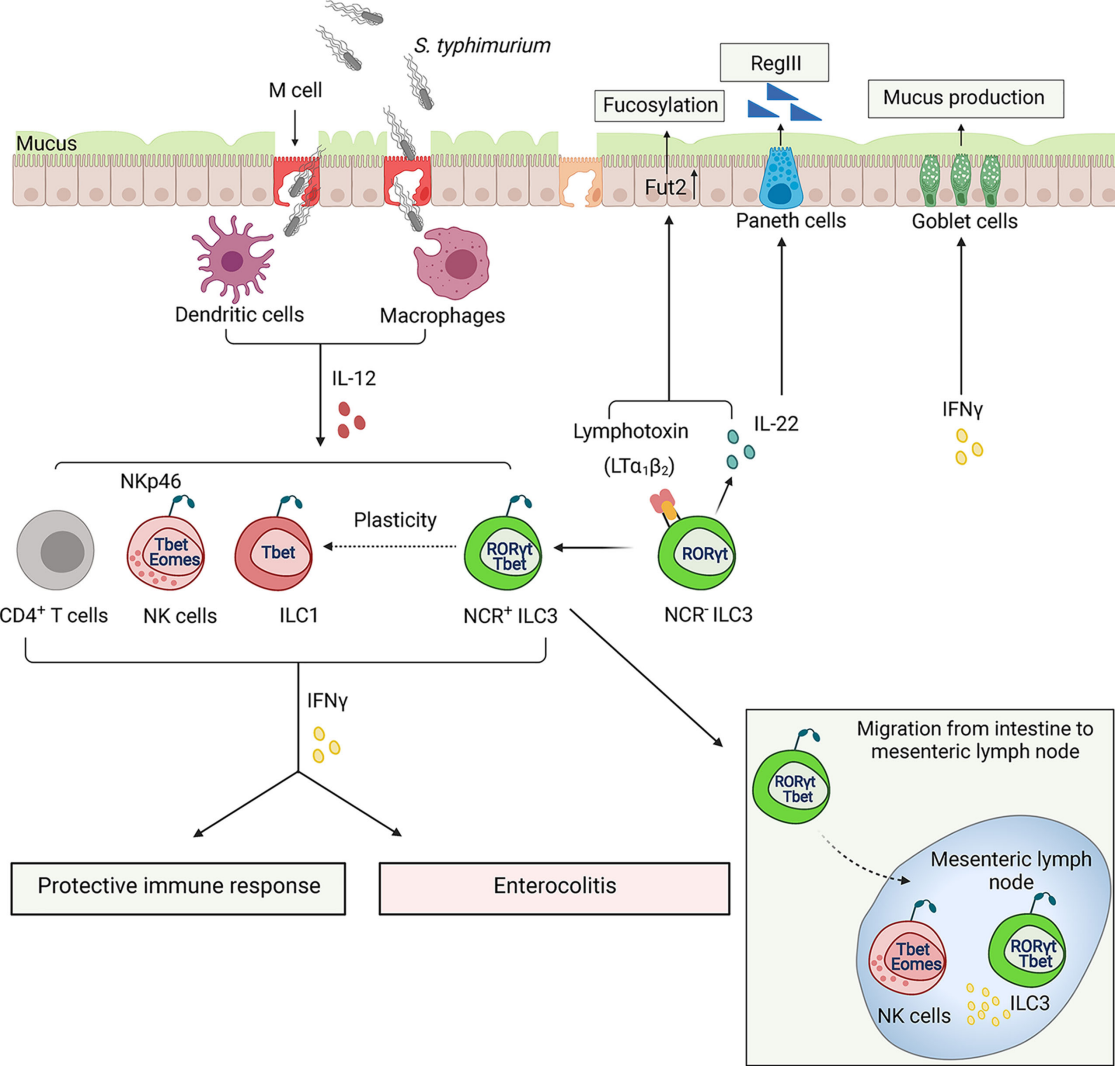
ILCs regulate the immune response to *Salmonella typhimurium*. *Salmonella* use microfold cells (M cells), intestinal epithelial cells as an entry point to invade the host. Following ingestion, bacterium enters lamina propria, where it encounters dendritic cells (DCs) and macrophages (MФ) which can spread bacteria to other tissues. Early control of *S. typhimurium* depends on the IFNγ production from activated ILCs and NK cells. *S. typhimurium* induces transition of NCR^-^RORγt^+^ILC3s to NCR^+^T-bet^+^ILC1s *via* NCR^+^T-bet^+^RORγt^+^ILC3s intermediates. IFNγ plays a dual role in pathogenesis of *Salmonella* infection: it stimulates bacteria clearance and activates mucus production but also induces tissue damage resulting in exacerbation of enterocolitis. A minor population of T-bet^+^ RORγt^+^ ILC3s can also migrate to mesenteric lymph node where ILC3s along with NK cells produce IFNγ. ILC3s can also contribute to protection against Salmonella *via* controlling fucosylation of epithelial cells by producing IL-22 and membrane LTα_1_LTβ_2_ lymphotoxin complex.

The innate immunity is essential for protection against *Salmonella* infection (Pham and McSorley, 2015). Recent data indicated that innate IFNγ controls bacterial loads in the small intestine and systemic bacterial dissemination (Songhet et al., 2011; Kupz et al., 2013). Interestingly, NKp46^+^T-bet^+^ILCs are the primary source of IFNγ, whereas only a small fraction of NK cells contributes to IFNγ production (Klose et al., 2013). Cell fate-mapping experiments revealed that the majority of NKp46^+^T-bet^+^ILCs have a history of RORγt expression, suggesting that *Salmonella* infection can induce transdifferentiation of NKp46^-^ILC3s to IFNγ-producing ILC1s (Klose et al., 2013) (**Figure 4**). T-bet is required for conversion of NKp46^+^RORγt^+^ILCs from NKp46^-^ILC3s because T-bet-deficient mice (*Tbx21^-/-^*) lack NKp46^+^T-bet^+^RORγt^+^ILCs and have fewer IFNγ-producing cells (Klose et al., 2013). IL-12 is required for IFNγ production by ILCs after *Salmonella* infection (Klose et al., 2013). Although IL-12^-/-^ mice have normal numbers of T-bet^+^RORγt^+^ ILCs, IFNγ production in these mice is impaired (Klose et al., 2013). These findings indicate that IFNγ production by NKp46^+^RORγt^+^ ILC3s is dependent on IL-12 and T-bet. Subsequent studies demonstrated that IFNγ production is dependent on the transcriptional complex of T-bet and Runx3 (Yin et al., 2018). Moreover, *Salmonella* infection induces Runx3 expression in ILC1s and NCR^+^ILC3s, but not in NK cells (Yin et al., 2018). Accordingly, genetic deletion of Runx3 in ILCs leads to high susceptibility of mice to *Salmonella* infection accompanied by decreased numbers of ILC1s and NCR^+^ILC3s (Yin et al., 2018). Although IFNγ produced by NKp46^+^RORγt^+^ ILCs contributes to bacterial clearance, it can also induce epithelial damage, as *Tbx21^-/-^* and *Ifngr1* ^-/-^ mice displayed reduced intestinal pathology 48h after infection (Klose et al., 2013). Additionally, IFNγ produced by mucosal NK cells and T cells can delay the resolution of intestinal inflammation by inducing STAT1 activation and blocking of IL-22-RegIIIβ-mediated antimicrobial defense (Dolowschiak et al., 2016). These studies suggest that IFNγ-producing ILCs can also contribute to intestinal pathology during *Salmonella* infection (**Figure 4**).

NCR^-^ILC3s contribute to the protection against *Salmonella* by producing IL-22, which in turn induces production of RegIIIβ and RegIIIγ antibacterial proteins by epithelial cells (Goto et al., 2014). Additionally, IL-22 stimulates expression of enzyme fucosyltransferase 2 (Fut2), which is required for epithelial fucosylation (Goto et al., 2014). Accordingly, RORγt^-/-^ mice and Fut2^-/-^ mice display an increased susceptibility to *Salmonella* infection (Goto et al., 2014). Interestingly, LTα^-/-^ mice and mice treated with LTβR-Ig inhibitor displayed reduced levels of Fut2 and fucosylation, suggesting that LTβR signaling is required for epithelial fucosylation (Goto et al., 2014). It is currently unclear whether LTβR signaling directly regulates Fut2-dependent fucosylation in epithelial cells or activates an indirect mechanism by inducing IL-22 production by ILC3s (**Figure 4**). LT expression by ILC3s is known to enhance IL-22 production by these cells *via* crosstalk with IL-23-producing mononuclear phagocytes and intestinal epithelial cells in response to extracellular bacterial pathogen *C. rodentium* or epithelial injury (Tumanov et al., 2011; Macho-Fernandez et al., 2015); however, it remains to be determined whether similar ILC3-LT-dependent mechanism is important for protection against *Salmonella*. A recent study demonstrated that LT expression by both ILC3s and B cells contributes to protection against *Salmonella* (Wroblewska et al., 2017). It was also found that LTβR^-/-^ mice display an impaired production of IFNγ in response to chronic *Salmonella* infection (Wroblewska et al., 2017). Whether LT controls IFNγ production by ILCs or adaptive immune cells remains to be determined.

Although ILCs are largely tissue-resident cells, there is emerging evidence that minor ILC populations can migrate to and within the tissues in response to the local environmental signals (Gasteiger et al., 2015; Kastele et al., 2021). A recent study showed that a small ILC population can migrate from the intestine to the mesenteric lymph node (MLN) in steady state and under inflammatory conditions (Kastele et al., 2021). Increased numbers of migratory RORγt^+^T-bet^+^ILCs were found in the lymph nodes of *Salmonella*-infected mice, which served as an early source of IFNγ along with NK cells (Kastele et al., 2021). However, the mechanism of ILC migration and its role in the regulation of adaptive immune response during inflammation remains poorly understood (**Figure 4**).

The major function of ILCs is to strengthen the epithelial barriers through various mechanisms. IFNγ was shown to induce production of mucins by goblet cells. Mucins form the inner mucus layer to protect epithelial cells from bacterial invasion (Songhet et al., 2011; Klose et al., 2013). A previous study demonstrated that MUC2-deficient mice showed an increased susceptibility to *Salmonella* infection (Zarepour et al., 2013). Fewer goblet cells filled with mucus were observed after infection with *Salmonella* (Klose et al., 2013). Moreover, *Tbx21* ^-/-^ mice, which lack IFNγ-producing Nkp46^+^RORγt^+^ILCs, showed a decrease in mucus secretion. Experiments with depletion of ILCs revealed reduced IFNγ and mucus secretion after *Salmonella* infection (Klose et al., 2013). These studies suggest that IFNγ-producing ILCs regulate goblet cells in response to *Salmonella* infection, although the detailed mechanism of ILC-dependent mucus secretion remains to be determined.

In summary, IFNγ-producing ILCs play a dual role in *Salmonella* infection. IFNγ production by ILCs is critical for bacterial clearance in the gut and to restore the epithelial barrier. However, IFNγ from ILCs can also induce mucus production and impair tight junction during infection, potentially leading to an additional tissue damage. Additional mechanistic studies are needed to dissect the role of ILC-produced IFNγ in maintaining the integrity of epithelial barrier during *Salmonella* infection.

### Yersinia enterocolitica

*Y. enterocolitica* and *Y. pseudotuberculosis* are food-borne enteropathogens which typically cause self-limiting infections of GI tract (Hering et al., 2011; Bancerz-Kisiel et al., 2018). However, in some cases these infections cause enteritis and mesenteric lymphadenitis. *Yersinia* invades intestinal barrier *via* M cells. After epithelial barrier invasion, bacteria replicates within PPs and then disseminates to the major lymphatic organs, particularly MLN in humans, as well as the spleen, liver, and lungs in rodents (Handley et al., 2005).

**Figure 5:**
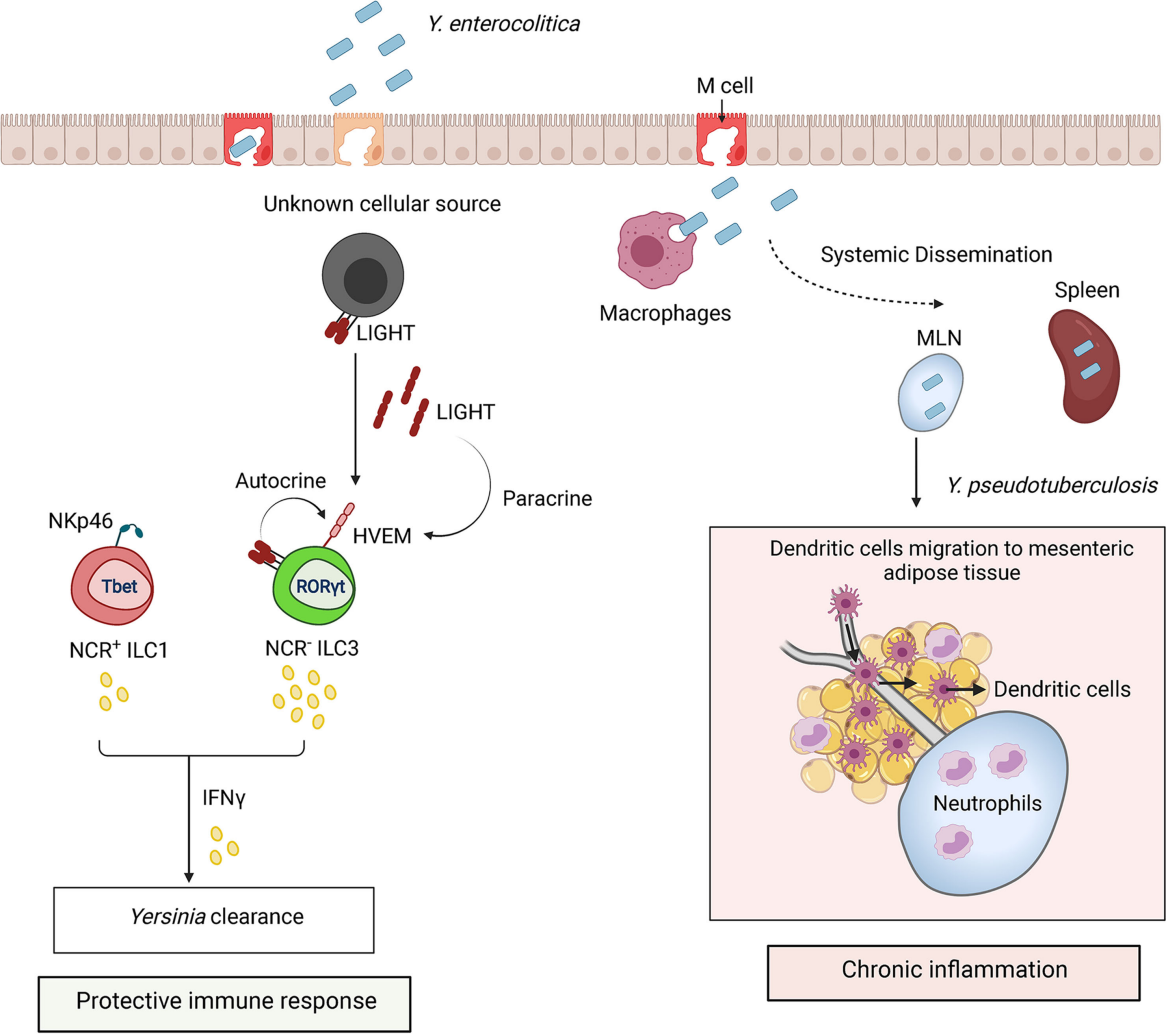
ILCs regulate the immune response to *Yersinia* infection. *Y. enterocolitica* and *Y. pseudotuberculosis* invade host through microfold cells (M cells). Bacteria replicate within Peyer’s patches after invasion and disseminate to mesenteric lymph nodes (MLN) and spleen. Protection against *Yersinia* is mediated by IFNγ production by ILC3s and ILC1s. HVEM signaling in NCR^-^ ILC3s by LIGHT activates IFNγ production. LIGHT is shown as a membrane bound form or secreted homotrimer. Sustained inflammation triggered by *Y. pseudotuberculosis* can lead to disruption of MLN lymphatics and accumulation of neutrophils which alter migration of DCs from MLN to mesenteric adipose tissue.

A recent study revealed protective role of ILC3s in early host response against *Y. enterocolitica* (Seo et al., 2018). Transfer of CCR6^-^ ILC3s, but not CCR6^+^ ILC3s, derived from small intestine lamina propria of naïve Rag1^-/-^ mice, rescued Rag2**^-/-^** Il2rg**^-/-^** mice from rapid weight loss caused by *Y. enterocolitica* infection (Seo et al., 2018). IFNγ, but not IL-17A or IL-22, was critical for mice survival (Seo et al., 2018). IFNγ is predominantly produced by NKp46^-^RORγt^+^ ILC3s and only a small fraction of ILC1s and NK cells after *Y. enterocolitica* infection (Seo et al., 2018) (**Figure 5**). It remains to be determined whether ILC3s undergo plasticity during *Y. enterocolitica* infection to produce IFNγ. Surprisingly, IFNγ production by ILC3s was dependent on LIGHT-HVEM signaling (Seo et al., 2018). Although the cellular source of LIGHT has not been identified in this study, it is known that all ILC subsets in small intestine express LIGHT and HVEM, thus both autocrine and paracrine mechanisms of HVEM activation are possible (Seo et al., 2018). Interestingly, *Y. pseudotuberculosis* infection is known to cause mesenteric lymphadenopathy due to disruption of lymphatic system, which results in compromised function of mucosal DCs shifted from MLN to adipose tissue (Fonseca et al., 2015). Therefore, future studies are needed to elucidate the role of ILCs in *Yersinia*-induced immunopathology in different tissues.

### *Chlamydia* Infection

*Chlamydiae* are obligate intracellular bacteria which can cause persistent infection in mammals and birds (Yeruva et al., 2013; Rank and Yeruva, 2014). Although in the majority of animals *Chlamydia* resides in the GI tract and infections occur *via* oral-fecal route (Yeruva et al., 2013; Rank and Yeruva, 2014), *Chlamydia trachomatis* is associated with human genital infection and is a leading cause of sexually transmitted bacterial diseases in humans (Vasilevsky et al., 2014; Zhong, 2021). Most chlamydial infections are asymptomatic and may persist in the genital tract for a long time. The data in humans suggest that *Chlamydia* can also establish an infection in the GI tract and lungs (Rank and Yeruva, 2014; Howe et al., 2019). *C. muridarum* is a mouse pathogen commonly used to study chlamydial genital infection. It also persistently colonizes the intestine (Igietseme et al., 2001; Yeruva et al., 2013). Gastrointestinal *Chlamydia* infection can spread to the genital tract, leading to genital tract pathology (Dai et al., 2016; Tian et al., 2020; Zhong, 2021). In contrast, intestinal *C. muridarum* infection can protect mice against subsequent genital tract infection, which is dependent on CD4^+^ T cells and B cells (Wang et al., 2018).

IFNγ is critical for protection against *Chlamydia* infection (Johansson et al., 1997; Perry et al., 1997; Mercado et al., 2021). Although IFNγ production by CD4^+^ T cells in response to *Chlamydia* infection has been described (Johansson et al., 1997; Perry et al., 1997), the role of distinct innate immune cell populations remains poorly defined.

The ILC-dependent protective mechanisms vary depending on the particular strain of *Chlamydia* and the route of infection. For example, IFNγ-producing NK cells are important for protection from genital *C. trachomatis* but not from pulmonary infection with *C. pneumonia* (Tseng and Rank, 1998; Rottenberg et al., 2000). Early studies demonstrated that IFNγ^+^ NK cells are recruited to the site of *C. trachomatis* infection as early as 12 hours after intravaginal inoculation of immunocompetent hosts and depletion of NK cells with anti-asialo-GM1 antibody led to impaired IFNγ production (Tseng and Rank, 1998). However, other studies showed that Rag1^-/-^ mice depleted of NK cells are resistant to *C. pheumoniae* (Rottenberg et al., 2000; Rottenberg et al., 2002). Since experimental strategies to deplete NK cells with anti-NK1.1 and anti-asialo-GM1 antibodies can also target ILC1s and ILC3s, the role of distinct ILC populations in immune response to *Chlamydia* remained unclear.

**Figure 6:**
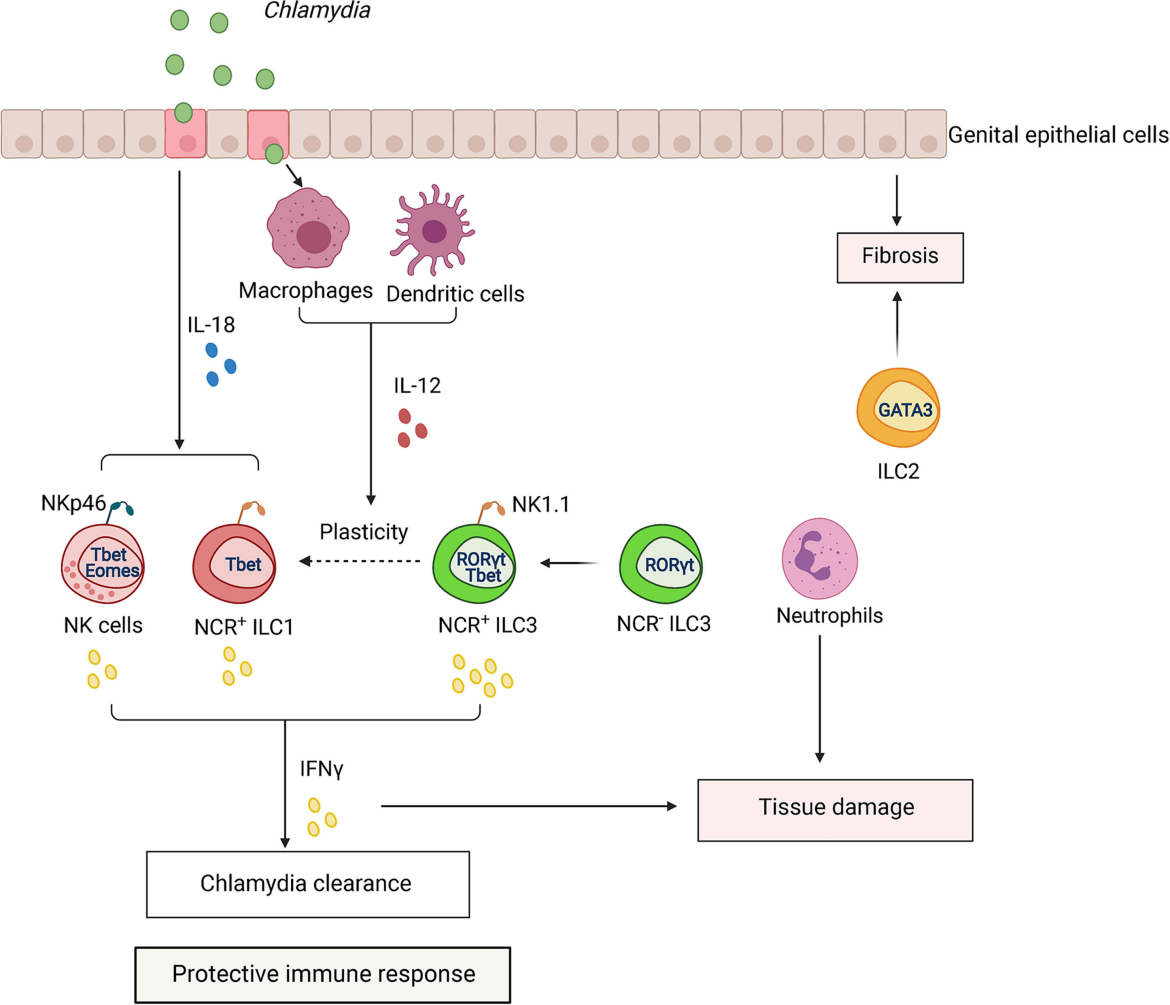
ILCs regulate the immune response to *Chlamydia* infection. *Chlamydia* colonizes epithelium of GI and genital tract. Infection activates dendritic cells (DCs) and macrophages (MФ), which produce IL-12. Activated epithelial cells produce IL-18 which synergizes with IL-12 to amplify IFNγ production by NK cells, ILC1s, and ILC3s, contributing to *Chlamydia* clearance. *Chlamydia* infection induces plasticity of RORγt^+^ ILC3s to IFNγ-producing ILC1s (ex-ILC3s). CCR2^+^ myeloid cells can induce expansion of ILCs in the tissue. Excessive activation of ILCs and infiltration of neutrophils can lead to tissue pathology.

Recent studies addressed the role of ILCs and NK cells in GI and genital tract *Chlamydia* infection (Koprivsek et al., 2020; Xu et al., 2020; Barth et al., 2021; He et al., 2021; Mercado et al., 2021) (**Figure 6**). A study using a *Chlamydia* mutant strain, which is unable to maintain long-lasting colonization in the GI tract, demonstrated that IFNγ-producing NK1.1^+^ILC3s can protect against colonization of the colon (Koprivsek et al., 2020). Furthermore, adoptive transfer of enriched ILC3s inhibited colonization of *C. muridarum* in the colon and restored IFNγ production in IL7R^-/-^ mice (He et al., 2021). Interestingly, it appears that NK cells are dispensable for the clearance of bacteria from the colon because adoptive transfer of RORγt^+^ILCs, but not RORγt^–^ILCs, restored colonization resistance of *Chlamydia* mutant in the colon (He et al., 2021). As some RORγt^+^ILCs expressed IFNγ, it was proposed that ex-ILC3s contribute to protection against *C. muridarum* mutant colonic colonization (He et al., 2021). Future cell fate mapping experiments will be required to test this hypothesis.

ILCs were also found to protect against *Chlamydia* in the genital tract (Xu et al., 2020; Mercado et al., 2021). Thus, Rag2**^-/-^** Il2rg**^-/-^** mice lacking all ILCs subsets succumbed earlier to genital *C. muridarum* infection compared to Rag1^-/-^ mice (Mercado et al., 2021). Increased bacterial titers were found in genital tracts of *C. trachomatis*-infected Rag2**^-/-^** Il2rg**^-/-^** mice in comparison with Rag1^-/-^ mice (Xu et al., 2020). Depletion of NK1.1^+^ cells in Rag1^-/-^ mice reduced IFNγ production and increased *C. trachomatis* titers in the endometrial tissue (Xu et al., 2020). NK1.1^+^ cell population contains a mixture of NK cells, ILC1s, and IFNγ-producing NK1.1^+^T-bet^+^RORγt^+^ILC3s. In line with this data, another study showed that ILCs can provide a source of IFNγ during *C. trachomatis* infection, as RORγt- and T-bet-deficient mice showed an increased bacterial burden in the genital tract (Xu et al., 2020). Additionally, depletion of NK1.1^+^ cells with anti-NK1.1. antibody reduced IFNγ production and increased *C. trachomatis* burden in the endometrial tissue (Xu et al., 2020). Recent comprehensive analysis of ILC populations by flow cytometry during the course of genital *C. muridarum* infection revealed that NK cells were the predominant ILCs, and they expanded 2-3 fold during infection (from ~50,000 cells in naïve mice to 150,000 cells by day 30 post infection) (Barth et al., 2021). ILC1s also expanded 5-10 fold (~4,000 cells in naïve mice to 20,000 cells by day 30). In contrast, ILC3 numbers remained very low throughout the infection (100-400 cells per genital tract). The genital tract also contained a small number of ILC2s (2000-3000 cells per genital tract). Furthermore, cell fate-mapping experiments revealed that only 10% of ILC1s had a history of RORγt expression (Barth et al., 2021). It remains unclear whether these very small numbers of ILC3s and ex-ILC3s could contribute to the protection against genital *Chlamydia* infection. The mechanism leading to specific expansion of ILC subsets in the genital tract remains to be determined and could potentially include proliferation of tissue-resident cells, recruitment, and differentiation. Although RORγt-deficient mice showed increased bacterial titers in the genital tract (Xu et al., 2020), other developmental defects in these mice, such as lack of lymph nodes and gut-associated lymphoid tissues could contribute to this phenotype. The role of ILC1s in protection is also unclear, as only about 25% of ILC1s produced IFNγ on day 4 after infection, but almost none did on day 30, although the accumulation of these cells in the genital tract continued until at least day 30 after infection (Barth et al., 2021). The role of ILC1s in *Chlamydia* infection remains contradictory, as NKp46^-/-^ mice did not display reduced numbers of ILC1s (Barth et al., 2021), in contrast to previous report (Wang et al., 2018). We speculate that the contribution of distinct populations of ILCs for protection vary between genital versus GI tract infection. It is possible that local cytokine microenvironment in GI tract influenced by signals from microbiota predominantly supports expansion of IFNγ-producing ex-ILC3s, whereas genital tract infection drives expansion of IFNγ-producing NK cells and ILC1s. As IFNγ-producing NK cells and ILC1s accumulate at mucosa at the same time post infection, it will be important to define whether these subsets play unique or redundant roles during infection. Further studies using more specific genetic targeting of distinct ILC populations are required to better define the specific contribution of ILC subsets to protection following GI and genital tract infections.

Genital tract infection is known to promote inflammation-associated scarring of the oviduct that leads to hydrosalpinx (Tang et al., 2013; Lei et al., 2014). The role of ILCs in tissue pathology remains poorly understood. Interestingly, oviduct weight as an indicator of pathology correlated with expansion of NK cells and ILC1s following genital *C. muridarum* infection (Barth et al., 2021). Myeloid cells can promote expansion of ILCs because CCR2^-/-^ mice displayed reduced numbers of ILCs in the genital tract (Barth et al., 2021). Additionally, depletion of neutrophils ameliorated tissue damage in genital *C. muridarum* infection (Zortel et al., 2018). Thus, interplay between ILCs and other immune cell populations can shape the outcome of *Chlamydia* infection (**Figure 6**). It will be important to define in future studies why some patients infected with *Chlamydia* develop pathology whereas others remain asymptomatic. A shift from Th1 to Th2 immune response was suggested to promote scarring and pathology development (Barth et al., 2021). As both ILC1s and ILC2s are present in the genital tract (Doisne et al., 2015), it is possible that ILC1s and NK cells predominantly contribute to bacterial clearance and host protection whereas ILC2s can induce inflammation and fibrosis.

### Mycobacterium tuberculosis

Tuberculosis disease has one of the highest fatality rates among human infections. The causative agent, *Mycobacterium tuberculosis* (*Mtb*), is a facultative mycobacterial pathogen which primarily resides in the phagosomes of macrophages (Podinovskaia et al., 2013). Ingestion of mycobacterium bacilli induces release of proinflammatory cytokines and chemokines to activate immune response. Early studies revealed an increased sensitivity of Rag2**^-/-^** Il2rg**^-/-^** mice to *Mtb* compared to Rag^-/-^ mice, which was attributed to the lack of IFNγ-producing NK cells in Rag2**^-/-^** Il2rg**^-/-^** mice (Feng et al., 2006). However, a recent study implicated the role of ILC3s in protective immunity to *Mtb* (Ardain et al., 2019a).

Although all ILC subsets were found in the airways, most studies were focused on ILC2s that were thought to be important for allergic airway inflammation (Halim et al., 2012; Yu et al., 2018; Xiao et al., 2021), chronic rhinosinusitis (Mjösberg et al., 2011), and asthma (McKenzie, 2014). While ILC2s are the predominant cells among ILCs in the mouse lungs in steady state, ILC3s are prevalent in human lung tissue in pulmonary disease (De Grove et al., 2016). During *Mtb* infection, ILCs become activated and accumulated in the lungs (Ardain et al., 2019a; Ardain et al., 2019b). While ILC2s accumulated at the later time points of *Mtb* infection in human and mouse lungs, ILC3s but not ILC1s rapidly accumulated in the lungs early during infection (Ardain et al., 2019a). Interestingly, the accumulation of alveolar macrophages coincided with an increase of ILC3s in the lungs (Ardain et al., 2019a). Moreover, mice lacking ILC3s exhibited reduced accumulation of alveolar macrophages and increased *Mtb* burden, suggesting that ILC3s contribute to early immune control of *Mtb* (Ardain et al., 2019a).

*Mtb* infection leads to type 1 immunity development with high levels of IFNγ production. One study showed that IFNγ^-/-^ mice and mice depleted of NK cells and NK1.1^+^ILC1s with anti-NK1.1 antibody did not show increased bacterial titers in the lungs, suggesting that IFNγ, NK cells and NK1.1^+^ILC1s are dispensable for control of early immunity to *Mtb* (Ardain et al., 2019a). Another study showed expansion of ILC1s in the lung as well as ILC1-like cells that expressed ILC1 markers T-bet, CD49a, and CD226 but were negative for NK1.1, NKp46, and Eomes (Corral et al., 2021) at later time points of infection. Furthermore, ILC1-like cells could originate from ILC2s during *Mtb* infection (Corral et al., 2021). Both ILC1s and ILC1-like cells contributed to IFNγ production (Corral et al., 2021). Moreover, protection against *Mtb* after BCG vaccination correlated with expansion of T-bet^+^ ILC1s and IFNγ production, indicating the protective role of ILC1s in mouse model (Steigler et al., 2018; Corral et al., 2021). Consistent with this data, recent study showed increased numbers of IFNγ-producing ILC1s in the blood of active tuberculosis patients (Pan et al., 2021).

Following pulmonary infection, activated immune cells infiltrate lungs, forming tertiary lymphoid structures known as inducible Bronchus-Associated Lymphoid Tissue (iBALT), which are important for generation of protective immune responses (Ulrichs et al., 2004; Day et al., 2010). Stromal cells express CXCL13 chemokine, which recruits lymphocytes in CXCR5-dependent manner, thereby inducing formation of iBALT (Marin et al., 2019; Zeng et al., 2020). In line with results obtained in mice, high levels of CXCL13 were detected in lungs of *Mtb* patients (Ardain et al., 2019a). Moreover, CXCR5- and CD103-expressing ILC3s were detected in *Mtb^+^* human lungs (Ardain et al., 2019a), however the mechanisms underlying migration of blood-resident ILCs to the lungs upon *Mtb* infection remain unknown.

IL-22 and IL-17 are the main effector cytokines produced by ILC3s (Vivier et al., 2018). Combined deletion of IL-22 and IL-17 affected the numbers of ILC3s, including CXCR5^+^ILC3s, but not ILC1s or ILC2s (Ardain et al., 2019a). Additionally, IL-22^-/-^/IL-17^-/-^ mice had higher *Mtb* burden in the lungs, suggesting that both IL-22 and IL-17 protect against *Mtb* infection (Ardain et al., 2019a). IL-23 and IL-1β activate production of IL-17 and IL-22 by ILC3s in the lungs (Cupedo et al., 2009; Ardain et al., 2019a). Moreover, it was shown that murine lung cells produce IL-23 in response to *Mtb*, and depletion of IL-23 leads to reduction of ILC3s and alveolar macrophages (Ardain et al., 2019a). Importantly, reduced accumulation of ILC3s in the lungs correlated with decreased formation of iBALT, indicating the role of ILC3s in formation of iBALT in a CXCL13-CXCR5-dependent manner. However, the precise mechanisms by which ILC3s contribute to formation of iBALT and protection against *Mtb* infection still needs to be defined.

Thus, accumulating evidence suggests that ILCs contribute to *Mtb* infection, and the tissue microenvironment may change ILCs phenotype and function during lung inflammation. The relative contribution of different subsets of ILCs to *Mtb* pathology remains unclear. Moreover, it remains to be determined whether targeting of specific ILC populations could have a therapeutic effect in *Mtb* patients. Further research is also needed to dissect the relative contribution of ILC subsets and T cells to disease.

### *Campylobacter* Infection

*Campylobacter* is a highly motile facultative bacteria causing the food-borne gastroenteritis called campylobacteriosis (Young et al., 2007). The main source of infection in humans is consumption of contaminated poultry where *Campylobacter* is a commensal. Among *Campylobacter* species, *C. jejuni* and *C. coli* are the major causes of acute gastroenteritis worldwide (Man, 2011; Kaakoush et al., 2015). In most cases, campylobacteriosis is self-limiting in healthy individuals; however, increasing evidence suggests that *Campylobacter* infection is associated with the development of autoimmune disorders affecting the nervous system and gastrointestinal tract (Ang et al., 2000; McCarthy and Giesecke, 2001; Nachamkin, 2002; Gradel et al., 2009). Moreover, patients with *C. jejuni* gastroenteritis are at a high risk of developing IBD (Navarro-Llavat et al., 2009; Antonelli et al., 2012; Arora et al., 2015). Since conventionally housed wild-type mice colonized with *C. jejuni* do not develop any adverse clinical signs or pathology (Mansfield et al., 2007; Lippert et al., 2009), most immunological studies of *Campylobacter* have been performed in mice with impaired IL-10 signaling. Oral inoculation of IL-10^-/-^ mice with *C. jejuni* leads to colon and cecum inflammation and immunohistopathological features which resemble campylobacteriosis in humans (Mansfield et al., 2007; Mansfield et al., 2008).

**Figure 7:**
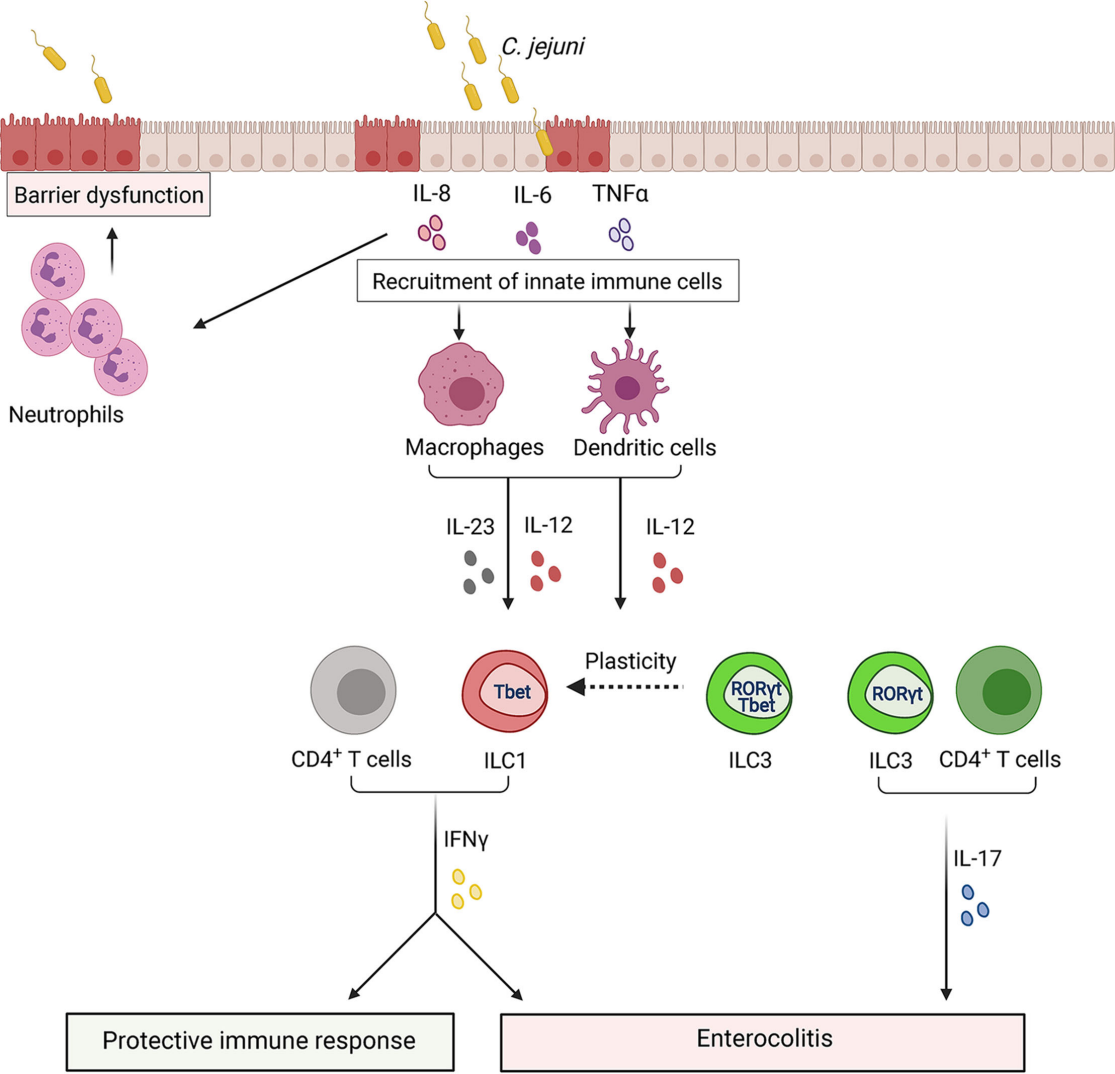
ILCs regulate the immune response to *Campylobacter jejuni*. Invasion of *Campylobacter* into epithelial cells leads to the damage of barrier structures. In response to damage, intestinal epithelial cells secrete cytokines to recruit monocytes, macrophages, dendritic cells. These cells release IL-23 and IL-12, leading to IFNγ production by ILCs at early stages of infection and by T cells at later stages. IFNγ can contribute to both protective and pathogenic responses to *C. jejuni*. IFNγ activates immune cells to help clear bacteria. Additionally, *C. jejuni* promotes conversion of ILC3s to IFNγ-producing ILC1s which contribute to intestinal pathology. IL-23 derived from antigen-presenting cells, such as dendritic cells, leads to IL-17 production by ILC3s and T cells which can also exacerbate colon inflammation.

*Campylobacter* colonizes ileum and colon, adhering to the mucus layer and invading epithelial cells. Upon bacterial invasion, epithelial cells secrete proinflammatory cytokines, including IL-8 and TNFα, to recruit innate immune cells for bacterial elimination at the site of the infection (Hameed, 2019) (**Figure 7**). Immune response to *Campylobacter* is characterized by the induction of mixed type 1 (IFNγ) and type 17 (IL-17A and IL-22) cytokine responses in both humans and mice (Edwards et al., 2010; Malik et al., 2014; Al-Banna et al., 2018). Both IFNγ and IL-17 were shown to contribute to *C. jejuni*-induced pathology (Malik et al., 2014; Muraoka et al., 2021). Analysis of cell types in the colon revealed that ILCs are the primary innate source of IFNγ in early onset of *C. jejuni*-mediated colitis (Muraoka et al., 2021). Moreover, it was shown that TCRβ/δ^-/-^ mice, which lack T cells, exhibit substantial intestinal inflammation in response to the infection (Muraoka et al., 2021). Depletion of CD4^+^ T cells did not reduce intestinal inflammation, demonstrating that CD4^+^ T cells are dispensable for colitis development in this model (Sun et al., 2012). Consistently, Rag2**^-/-^** Il2rg**^-/-^** mice were protected from colitis, suggesting the role of ILCs in intestinal pathology at an early onset of the disease (Muraoka et al., 2021). Neutralization of IFNγ in Rag2^-/-^Il2rg^-/-^ mice also reduced bacterial burden in the colon compared to Rag2^-/-^ mice, suggesting that IFNγ- producing ILCs are dispensable for protection against *C. jejuni*. Additionally, neutralization of IFNγ reduced intestinal disease in IL-10^-/-^TCRβ/δ^-/-^ and IL-10^-/-^ mice (Malik et al., 2014; Muraoka et al., 2021). These studies suggest that IFNγ produced by ILCs contributes to *C. jejuni*-induced intestinal pathology but is dispensable for protection.

The role of IFNγ in human campylobacteriosis remains unclear. Earlier studies demonstrated that IFNγ can enhance killing of *C jejuni* by macrophages (Wassenaar et al., 1997). The severity of disease was lower in healthy volunteers who exhibited increased IFNγ production by peripheral blood mononuclear cells stimulated with heat-killed *C. jejuni* before experimental infection (Tribble et al., 2010). However, another study did not find a difference in the amount of *C. jejuni*-specific IFNγ^+^CD4^+^ T cells between protected and unprotected subjects (Fimlaid et al., 2014). Development of animal models of *C. jejuni*-induced disease using mice reconstituted with human immune system will help to better understand the role of IFNγ and ILCs in human disease.

Expression of natural cytotoxicity receptors (NK1.1^+^ and NKp46^+^) by ILC1s and ILC3s has been associated with the production of IFNγ (Klose et al., 2013). However, growing evidence suggests that NK1.1^-^ILCs represent the primary source of IFNγ during inflammation. Analysis of ILCs in the colon during *C. jejuni* infection revealed that NK1.1^-^ILCs were the main innate source of IFNγ (Muraoka et al., 2021). Some IFNγ-producing ILCs can arise from ILC3s that downregulate RORγt and upregulate T-bet expression (ex-ILC3s) (**Figure 7**) (Klose et al., 2013; Vivier et al., 2018). RORγt cell fate-mapping experiments revealed that the majority of IFNγ-producing ILCs had a history of RORγt expression, indicating that *C. jejuni* infection facilitates conversion of ILC3s to ILC1s (Muraoka et al., 2021). The mechanism leading to ILC3>ILC1 plasticity during *C. jejuni* infection remains poorly understood. ILC3s>ILC1s plasticity was observed in response to IL-12 in human samples (Bernink et al., 2015). IL-12 levels in the colon are increased during *C. jejuni* infection (Edwards et al., 2010; Malik et al., 2014; Muraoka et al., 2021). Therefore, it is possible that IL-12 regulates the plasticity of ILCs. However, additional studies are required to test this hypothesis.

*C. jejuni* infection enhances the production of IL-17, IL-22 and IL-23 (Malik et al., 2014; Muraoka et al., 2021). Monocyte-derived dendritic cells from human PBMCs showed upregulation of both IL-17 and IL-22 cytokines after *C. jejuni* infection (Edwards et al., 2010). A recent study revealed IL-23 as a critical driver of inflammation during *C. jejuni* infection (Jing et al., 2020). Additionally, previous studies showed the pathogenic role of IL-23 in other models of infectious colitis (Kullberg et al., 2006; Buonomo et al., 2013). IL-23 is a well-known inducer of IL-22 and IL-17. Consistently, IL-23-deficient mice displayed reduced intestinal pathology and low levels of IFNγ, IL-17 and IL-22 in the colon following *C. jejuni* infection (Jing et al., 2020). However, in contrast to IFNγ and IL-17, abrogation of IL-22 signaling in mice with impaired IL-10 signaling did not have impact on *C. jejuni*-induced colitis and bacterial clearance (Jing et al., 2020). Interestingly, in another study in IL-10-sufficient mice, IL-22 deficiency led to increased bacterial burden in the colon (Heimesaat et al., 2016), suggesting that differences in intestinal microenvironment can affect *C. jejuni* colonization and disease development. Furthermore, IL-23 induced IL-17 and IFNγ by ILC1s and ILC3s, promoting colon inflammation during the early stage of *C. jejuni* infection (**Figure 7**) (Jing et al., 2020). IL-17 is also known as an important activator of innate immune responses that induce neutrophil recruitment (Pappu et al., 2012). However, neutrophils can also contribute to intestinal pathology during *C. jejuni* infection (Sun et al., 2012).

Together, these studies demonstrate that ILCs are critical for the initiation of inflammatory immune responses against *C. jejuni.* Moreover, ILC3s undergo transdifferentiation to ILC1s during infection. However, there are many critical questions that remain to be addressed. Whether ILC subsets contribute to intestinal pathology in human campylobacteriosis? Whether effects of ILCs in immune response depend on the *Campylobacter* strain and immunological status of the host? Additionally, host microbiota can also regulate ILCs functions and cooperation of ILCs with other immune cells (Mortha et al., 2014). Therefore, understanding the role of microbiota in activation and plasticity of ILCs during *C. jejuni* infection will better define mechanisms of *C. jejuni*-induced intestinal pathology.

## Conclusions

Over the last ten years research has identified ILCs as a heterogeneous family of innate immune cells that have very diverse functions in different barrier tissues. The precise role of ILCs is context-dependent and is regulated by tissue microenvironment. The environmental factors, including commensal microbiota and pathogens, can influence immune homeostasis and regulate plasticity and functions of ILCs. Although ILCs comprise a relatively minor cell population compared to other immune cell types, it is now well appreciated that while ILCs are essential for the maintenance of tissue homeostasis and elimination of pathogens, they can also contribute to chronic inflammation and tissue pathology.

Despite significant progress that has been made in understanding the function of ILCs in homeostatic conditions and during infection, many questions still remain. Under inflammatory conditions cytokines and tissue factors can change ILC composition. Depending on the type of signal, ILCs can transdifferentiate and change the outcome of the disease. Accordingly, both ILC1s and ILC3s can produce IFNγ under inflammatory conditions, which may have different effects during acute and chronic infections. It is important to study the relative functions of specific ILC subsets under physiological and inflammatory conditions because different subsets of ILCs can be protective or pathogenic (**Table 1**). Additional studies aimed to characterize the effects of environmental cues, such as dietary factors, metabolites and inflammatory mediators, on ILC development and function, will further advance our understanding of ILCs contribution to the pathogenesis of infectious diseases.

**Table 1:** The role of ILCs in intracellular pathogen infection.

Pathogen	ILC subset	Tissue	Cytokine	Role	References
*Toxoplasma gondii*	NK, ILC1	Small intestine	IFNγ	Protection	(Klose et al., 2014; Lopez-Yglesias et al., 2021)
	ILC3		IL-22	Pathology	(Munoz et al., 2015)
*Salmonella typhimurium*	ILC1, NCR^+^ILC3	Large intestine	IFNγ	Dual role	(Klose et al., 2013; Yin et al., 2018)
*Listeria monocytogenes*	NK, ILC1, NCR^+^ILC3	Small intestine	IFNγ	Protection	(Reynders et al., 2011; Yin et al., 2018; Dulson et al., 2019)
*Yersinia enterocolitica*	NCR^-^ILC3	Small intestine	IFNγ	Protection	(Seo et al., 2018)
*Chlamydia muridarum*	NCR^+^ILC3	Large intestine	IFNγ	Protection	(Koprivsek et al., 2020; He et al., 2021)
*Chlamydia trachomatis*	NK, ILC1, NCR^+^ILC3	Genital tract	IFNγ	Protection	(Xu et al., 2020; Barth et al., 2021)
*Mycobacterium tuberculosis*	ILC3 ILC1	Lungs	IL-22/IL-17 IFNγ	Protection Protection	(Ardain et al., 2019) (Corral et al., 2021; Pan et al., 2021)
*Campylobacter jejuni*	ILC1	Large intestine	IFNγ	Dual role	(Muraoka et al., 2021)
	NCR^-^ILC3		IFNγ/IL-17		(Jing et al., 2020)
*Plasmodium berghei*	ILC2	Spleen	IL-4/ IL-5/IL-13	Protection	(Besnard et al., 2015)

Although recent progress has revealed the role of ILCs in shaping the adaptive immune response by direct cell contact or by cytokine production, a lot more is still to be explored to uncover the function of ILCs in regulating the adaptive immune response following different infections in various tissues. For example, it is still unclear how ILCs regulate Th1 and Th17 immune responses during inflammation, and whether these mechanisms are context-dependent. Identification of mechanisms by which ILCs regulate the adaptive immune response across different infections will provide insights into the specific factors that may control the disease initiation and progression. The role of ILCs in protection and pathogenesis of human infections still needs to be better defined.

Over the last few years many small molecules and monoclonal antibodies that potentially may directly or indirectly target effector functions of ILCs, have been developed, such as JAK inhibitors (Schwartz et al., 2017; Robinette et al., 2018), TNF inhibitors, vedolizumab (anti-α4β7 integrin mAb) (Cobb and Verneris, 2021) and ustekinumab (IL-12/23p40 mAb) (Creyns et al., 2020; Cobb and Verneris, 2021). However, their selectivity and effects on ILCs are not fully characterized and need to be proven. Whether the application of these molecules will be beneficial for targeting ILCs in infectious diseases needs to be determined. Future work should focus on understanding the protective and pathogenic mechanisms of ILCs in different infections combining knowledge obtained from mouse models and human studies. These findings could uncover more specific targets for disease treatment.

## Author Contributions

Study concept and design: AK and AT. Wrote and edited manuscript: AK, EK, and AT. All authors contributed to the article and approved the submitted version.

## Funding

This research was supported by grants from NIH (AI135574, NS112263). AT was supported by the Max and Minnie Tomerlin Voelcker Fund, and William and Ella Owens Medical Research Foundation.

## Conflict of Interest

The authors declare that the research was conducted in the absence of any commercial or financial relationships that could be construed as a potential conflict of interest.

## Publisher’s Note

All claims expressed in this article are solely those of the authors and do not necessarily represent those of their affiliated organizations, or those of the publisher, the editors and the reviewers. Any product that may be evaluated in this article, or claim that may be made by its manufacturer, is not guaranteed or endorsed by the publisher.

## Abbreviations

ILCs, innate lymphoid cells; NK cells, natural killer cells; Th1, type 1 T helper cells; Th0, naïve T cells; IFNγ, interferon gamma; NCR, natural cytotoxicity receptor; T-bet, the interferon-γ inducible T-box transcription factor; RORγt, retinoic acid-related orphan receptor γt; M1, M1 type macrophage; iNOS, inducible nitric oxide synthase; ROS, reactive oxygen species; APC, antigen-presenting cell; MHC, major histocompatibility complex; LTα_1_LTβ_2_, heterotrimeric lymphotoxin complex; CCL2, C-C motif ligand 2; CXCL10/11, C-X-C motif chemokine ligand 10/11; LTβR, lymphotoxin beta receptor; LT, lymphotoxin; LIGHT, lymphotoxin-like inducible protein that competes with glycoprotein D for herpes virus entry on T cells; HVEM, herpes virus entry mediator; MLN, mesenteric lymph node; IDO, indoleamine 2,3-dioxygenase; iBALT, inducible bronchus-associated lymphoid tissue; *T. gondii, Toxoplasma gondii; C. jejuni, Campylobacter jejuni; Y. enterocolitica*, *Yersinia enterocolitica; Mtb, Mycobacterium tuberculosis*; *C. muridarum, Chlamydia muridarum; C. rodentium*, *Citrobacter rodentium*.

## References

[B1] Ahmad R. Sorrell M. F. Batra S. K. Dhawan P. Singh A. B. (2017). Gut Permeability and Mucosal Inflammation: Bad, Good or Context Dependent. Mucosal Immunol. 10, 307–317. doi: 10.1038/mi.2016.128 28120842 PMC6171348

[B2] Al-Banna N. A. Cyprian F. Albert M. J. (2018). Cytokine Responses in Campylobacteriosis: Linking Pathogenesis to Immunity. Cytokine Growth Factor Rev. 41, 75–87. doi: 10.1016/j.cytogfr.2018.03.005 29550265

[B3] Amaldi I. Reith W. Berte C. Mach B. (1989). Induction of Hla Class Ii Genes by Ifn-Gamma is Transcriptional and Requires a Trans-Acting Protein. J. Immunol. (Baltimore Md. 1950) 142(3), 999–1004. 2492334

[B4] Ang C. W. van Doorn P. A. Endtz H. P. Merkies I. S. Jacobs B. C. de Klerk M. A.. (2000). A Case of Guillain-Barre Syndrome Following a Family Outbreak of Campylobacter Jejuni Enteritis. J. Neuroimmunol. 111, 229–233. doi: 10.1016/s0165-5728(00)00369-6 11063843

[B5] Antonelli E. Baldoni M. Giovenali P. Villanacci V. Essatari M. Bassotti G. (2012). Intestinal Superinfections in Patients With Inflammatory Bowel Diseases. J. Crohns Colitis 6, 154–159. doi: 10.1016/j.crohns.2011.07.012 22325169

[B6] Ardain A. Domingo-Gonzalez R. Das S. Kazer S. W. Howard N. C. Singh A.. (2019a). Group 3 Innate Lymphoid Cells Mediate Early Protective Immunity Against Tuberculosis. Nature 570, 528–532. doi: 10.1038/s41586-019-1276-2 31168092 PMC6626542

[B7] Ardain A. Porterfield J. Z. Kloverpris H. N. Leslie A. (2019b). Type 3 Ilcs in Lung Disease. Front. Immunol. 10, 92. doi: 10.3389/fimmu.2019.00092 30761149 PMC6361816

[B8] Arora Z. Mukewar S. Wu X. Shen B. (2015). Risk Factors and Clinical Implication of Superimposed Campylobacter Jejuni Infection in Patients With Underlying Ulcerative Colitis. Gastroenterol. Rep. 4, 287–292. doi: 10.1093/gastro/gov029 PMC5193056 26159630

[B9] Bal S. M. Bernink J. H. Nagasawa M. Groot J. Shikhagaie M. M. Golebski K.. (2016). 1β, Il-4 and Il-12 Control the Fate of Group 2 Innate Lymphoid Cells in Human Airway Inflammation in the Lungs. Nat. Immunol. 17, 636–645. doi: 10.1038/ni.3444 27111145

[B10] Bal S. M. Golebski K. Spits H. (2020). Plasticity of Innate Lymphoid Cell Subsets. Nat. Rev. Immunol. 20, 552–565. doi: 10.1038/s41577-020-0282-9 32107466

[B11] Bancerz-Kisiel A. Pieczywek M. Łada P. Szweda W. (2018). The Most Important Virulence Markers of Yersinia Enterocolitica and Their Role During Infection. Genes 9, 235. doi: 10.3390/genes9050235 PMC5977175 29751540

[B12] Bando J. K. Gilfillan S. Di Luccia B. Fachi J. L. Sécca C. Cella M.. (2020). Ilc2s are the Predominant Source of Intestinal Ilc-Derived Il-10. J. Exp. Med. 217 (2), e20191520. doi: 10.1084/jem.20191520 31699824 PMC7041711

[B13] Bar-Ephraïm Y. E. Mebius R. E. (2016). Innate Lymphoid Cells in Secondary Lymphoid Organs. Immunol. Rev. 271, 185–199. doi: 10.1111/imr.12407 27088915

[B14] Barth S. Kirschnek S. Ortmann N. Tanriver Y. Häcker G. (2021). The Reaction of Innate Lymphoid Cells in the Mouse Female Genital Tract to Chlamydial Infection. Infect. Immun. 89 (11), Iai0080020. doi: 10.1128/iai.00800-20 PMC8519299 34424753

[B15] Beck K. Ohno H. Satoh-Takayama N. (2020). Innate Lymphoid Cells: Important Regulators of Host-Bacteria Interaction for Border Defense. Microorganisms 8 (9), 1342. doi: 10.3390/microorganisms8091342 PMC7563982 32887435

[B16] Bernink J. H. Krabbendam L. Germar K. de Jong E. Gronke K. Kofoed-Nielsen M.. (2015). Interleukin-12 and -23 Control Plasticity of Cd127(+) Group 1 and Group 3 Innate Lymphoid Cells in the Intestinal Lamina Propria. Immunity 43, 146–160. doi: 10.1016/j.immuni.2015.06.019 26187413

[B17] Bernink J. H. Peters C. P. Munneke M. te Velde A. A. Meijer S. L. Weijer K.. (2013). Human Type 1 Innate Lymphoid Cells Accumulate in Inflamed Mucosal Tissues. Nat. Immunol. 14, 221–229. doi: 10.1038/ni.2534 23334791

[B18] Besnard A. G. Guabiraba R. Niedbala W. Palomo J. Reverchon F. Shaw T. N.. (2015). Il-33-Mediated Protection Against Experimental Cerebral Malaria is Linked to Induction of Type 2 Innate Lymphoid Cells, M2 Macrophages and Regulatory T Cells. PloS Pathog. 11, e1004607. doi: 10.1371/journal.ppat.1004607 25659095 PMC4450060

[B19] Bielecki P. Riesenfeld S. J. Hütter J.-C. Torlai Triglia E. Kowalczyk M. S. Ricardo-Gonzalez R. R.. (2021). Skin-Resident Innate Lymphoid Cells Converge on a Pathogenic Effector State. Nature 592, 128–132. doi: 10.1038/s41586-021-03188-w 33536623 PMC8336632

[B20] Blumenthal A. Nagalingam G. Huch J. H. Walker L. Guillemin G. J. Smythe G. A.. (2012). M. Tuberculosis Induces Potent Activation of Ido-1, But This is Not Essential for the Immunological Control of Infection. PloS One 7, e37314. doi: 10.1371/journal.pone.0037314 22649518 PMC3359358

[B21] Bogdan C. (2015). Nitric Oxide Synthase in Innate and Adaptive Immunity: An Update. Trends Immunol. 36, 161–178. doi: 10.1016/j.it.2015.01.003 25687683

[B22] Braverman J. Stanley S. A. (2017). Nitric Oxide Modulates Macrophage Responses to Mycobacterium Tuberculosis Infection Through Activation of Hif-1α and Repression of Nf-κb. J. Immunol. (Baltimore Md. 1950) 199, 1805–1816. doi: 10.4049/jimmunol.1700515 PMC5568107 28754681

[B23] Brüggen M. C. Bauer W. M. Reininger B. Clim E. Captarencu C. Steiner G. E.. (2016). *In Situ* Mapping of Innate Lymphoid Cells in Human Skin: Evidence for Remarkable Differences Between Normal and Inflamed Skin. J. Invest. Dermatol. 136, 2396–2405. doi: 10.1016/j.jid.2016.07.017 27456756

[B24] Buonocore S. Ahern P. P. Uhlig H. H. Ivanov I. I. Littman D. R. Maloy K. J.. (2010). Innate Lymphoid Cells Drive Interleukin-23-Dependent Innate Intestinal Pathology. Nature 464, 1371–1375. doi: 10.1038/nature08949 20393462 PMC3796764

[B25] Buonomo E. L. Madan R. Pramoonjago P. Li L. Okusa M. D. Petri W. A. Jr. (2013). Role of Interleukin 23 Signaling in Clostridium Difficile Colitis. J. Infect. Dis. 208, 917–920. doi: 10.1093/infdis/jit277 23776194 PMC3749013

[B26] Cardoso V. Chesné J. Ribeiro H. García-Cassani B. Carvalho T. Bouchery T.. (2017). Neuronal Regulation of Type 2 Innate Lymphoid Cells via Neuromedin U. Nature 549, 277–281. doi: 10.1038/nature23469 28869974 PMC5714273

[B27] Casadevall A. Fang F. C. (2020). The Intracellular Pathogen Concept. Mol. Microbiol. 113, 541–545. doi: 10.1111/mmi.14421 31762116

[B28] Castellanos J. G. Woo V. Viladomiu M. Putzel G. Lima S. Diehl G. E.. (2018). Microbiota-Induced Tnf-Like Ligand 1a Drives Group 3 Innate Lymphoid Cell-Mediated Barrier Protection and Intestinal T Cell Activation During Colitis. Immunity 49, 1077–1189.e5. doi: 10.1016/j.immuni.2018.10.014 30552020 PMC6301104

[B29] Cayrol C. Girard J. P. (2019). Innate Lymphoid Cells in Asthmatic Patients. J. Allergy Clin. Immunol. 143, 1739–1741. doi: 10.1016/j.jaci.2019.03.011 30926530

[B30] Chelakkot C. Ghim J. Ryu S. H. (2018). Mechanisms Regulating Intestinal Barrier Integrity and its Pathological Implications. Exp. Mol. Med. 50, 1–9. doi: 10.1038/s12276-018-0126-x PMC6095905 30115904

[B31] Chen W. Lai D. Li Y. Wang X. Pan Y. Fang X.. (2021). Neuronal-Activated Ilc2s Promote Il-17a Production in Lung γδ T Cells During Sepsis. Front. Immunol. 12, 670676. doi: 10.3389/fimmu.2021.670676 33995408 PMC8119647

[B32] Chua H. H. Chou H. C. Tung Y. L. Chiang B. L. Liao C. C. Liu H. H.. (2018). Intestinal Dysbiosis Featuring Abundance of Ruminococcus Gnavus Associates With Allergic Diseases in Infants. Gastroenterology 154, 154–167. doi: 10.1053/j.gastro.2017.09.006 28912020

[B33] Chun E. Lavoie S. Fonseca-Pereira D. Bae S. Michaud M. Hoveyda H. R.. (2019). Metabolite-Sensing Receptor Ffar2 Regulates Colonic Group 3 Innate Lymphoid Cells and Gut Immunity. Immunity 51, 871–884.e6. doi: 10.1016/j.immuni.2019.09.014 31628054 PMC6901086

[B34] Clark J. T. Christian D. A. Gullicksrud J. A. Perry J. A. Park J. Jacquet M.. (2021). Il-33 Promotes Innate Lymphoid Cell-Dependent Ifn-γ Production Required for Innate Immunity to Toxoplasma Gondii. eLife 10, e65614. doi: 10.7554/eLife.65614 33929319 PMC8121546

[B35] Cobb L. M. Verneris M. R. (2021). Therapeutic Manipulation of Innate Lymphoid Cells. JCI Insight 6(6), e146006. doi: 10.1172/jci.insight.146006 PMC8026185 33749662

[B36] Colonna M. (2009). Interleukin-22-Producing Natural Killer Cells and Lymphoid Tissue Inducer-Like Cells in Mucosal Immunity. Immunity 31, 15–23. doi: 10.1016/j.immuni.2009.06.008 19604490

[B37] Corral D. Charton A. Krauss M. Z. Blanquart E. Levillain F. Lefrançais E.. (2021). Metabolic Control of Type 2 Innate Lymphoid Cells Plasticity Toward Protective Type 1-Like Cells During Mycobacterium Tuberculosis Infection. bioRxiv. doi: 10.1101/2021.01.19.427257

[B38] Couturier-Maillard A. Froux N. Piotet-Morin J. Michaudel C. Brault L. Le Bérichel J.. (2018). Interleukin-22-Deficiency and Microbiota Contribute to the Exacerbation of Toxoplasma Gondii-Induced Intestinal Inflammation. Mucosal Immunol. 11, 1181–1190. doi: 10.1038/s41385-018-0005-8 29728643

[B39] Crellin N. K. Trifari S. Kaplan C. D. Satoh-Takayama N. Di Santo J. P. Spits H. (2010). Regulation of Cytokine Secretion in Human Cd127(+) Lti-Like Innate Lymphoid Cells by Toll-Like Receptor 2. Immunity 33, 752–764. doi: 10.1016/j.immuni.2010.10.012 21055975

[B40] Creyns B. Jacobs I. Verstockt B. Cremer J. Ballet V. Vandecasteele R.. (2020). Biological Therapy in Inflammatory Bowel Disease Patients Partly Restores Intestinal Innate Lymphoid Cell Subtype Equilibrium. Front. Immunol. 11, 1847. doi: 10.3389/fimmu.2020.01847 32983101 PMC7481382

[B41] Cupedo T. Crellin N. K. Papazian N. Rombouts E. J. Weijer K. Grogan J. L.. (2009). Human Fetal Lymphoid Tissue-Inducer Cells are Interleukin 17-Producing Precursors to Rorc+ Cd127+ Natural Killer-Like Cells. Nat. Immunol. 10, 66–74. doi: 10.1038/ni.1668 19029905

[B42] Dai J. Zhang T. Wang L. Shao L. Zhu C. Zhang Y.. (2016). Intravenous Inoculation With Chlamydia Muridarum Leads to a Long-Lasting Infection Restricted to the Gastrointestinal Tract. Infect. Immun. 84, 2382–2388. doi: 10.1128/IAI.00432-16 27271744 PMC4962645

[B43] Darwich L. Coma G. Peña R. Bellido R. Blanco E. J. Este J. A.. (2009). Secretion of Interferon-Gamma by Human Macrophages Demonstrated at the Single-Cell Level After Costimulation With Interleukin (Il)-12 Plus Il-18. Immunology 126, 386–393. doi: 10.1111/j.1365-2567.2008.02905.x 18759749 PMC2669819

[B44] Daussy C. Faure F. Mayol K. Viel S. Gasteiger G. Charrier E.. (2014). T-Bet and Eomes Instruct the Development of Two Distinct Natural Killer Cell Lineages in the Liver and in the Bone Marrow. J. Exp. Med. 211, 563–577. doi: 10.1084/jem.20131560 24516120 PMC3949572

[B45] Day T. A. Koch M. Nouailles G. Jacobsen M. Kosmiadi G. A. Miekley D.. (2010). Secondary Lymphoid Organs are Dispensable for the Development of T-Cell-Mediated Immunity During Tuberculosis. Eur. J. Immunol. 40, 1663–1673. doi: 10.1002/eji.201040299 20222088

[B46] Decker T. Stockinger S. Karaghiosoff M. Müller M. Kovarik P. (2002). Ifns and Stats in Innate Immunity to Microorganisms. J. Clin. Invest. 109, 1271–1277. doi: 10.1172/jci15770 12021240 PMC150987

[B47] De Grove K. C. Provoost S. Verhamme F. M. Bracke K. R. Joos G. F. Maes T.. (2016). Characterization and Quantification of Innate Lymphoid Cell Subsets in Human Lung. PloS One 11, e0145961. doi: 10.1371/journal.pone.0145961 26727464 PMC4699688

[B48] Denkers E. Y. Gazzinelli R. T. Martin D. Sher A. (1993). Emergence of Nk1.1+ Cells as Effectors of Ifn-Gamma Dependent Immunity to Toxoplasma Gondii in Mhc Class I-Deficient Mice. J. Exp. Med. 178, 1465–1472. doi: 10.1084/jem.178.5.1465 8228800 PMC2191244

[B49] Doisne J. M. Balmas E. Boulenouar S. Gaynor L. M. Kieckbusch J. Gardner L.. (2015). Composition, Development, and Function of Uterine Innate Lymphoid Cells. J. Immunol. (Baltimore Md. 1950) 195, 3937–3945. doi: 10.4049/jimmunol.1500689 PMC4592103 26371244

[B50] Dolowschiak T. Mueller A. A. Pisan L. J. Feigelman R. Felmy B. Sellin M. E.. (2016). Ifn-γ Hinders Recovery From Mucosal Inflammation During Antibiotic Therapy for Salmonella Gut Infection. Cell Host Microbe 20, 238–249. doi: 10.1016/j.chom.2016.06.008 27453483

[B51] Dulson S. J. Watkins E. E. Crossman D. K. Harrington L. E. (2019). Stat4 Directs a Protective Innate Lymphoid Cell Response to Gastrointestinal Infection. J. Immunol. (Baltimore Md. 1950) 203, 2472–2484. doi: 10.4049/jimmunol.1900719 PMC6810903 31562212

[B52] Dunay I. R. Damatta R. A. Fux B. Presti R. Greco S. Colonna M.. (2008). Gr1(+) Inflammatory Monocytes are Required for Mucosal Resistance to the Pathogen Toxoplasma Gondii. Immunity 29, 306–317. doi: 10.1016/j.immuni.2008.05.019 18691912 PMC2605393

[B53] Dunay I. R. Diefenbach A. (2018). Group 1 Innate Lymphoid Cells in Toxoplasma Gondii Infection. Parasite Immunol. 40(2), e12516. doi: 10.1111/pim.12516 29315653

[B54] Eberl G. Marmon S. Sunshine M. J. Rennert P. D. Choi Y. Littman D. R. (2004). An Essential Function for the Nuclear Receptor Rorgamma(T) in the Generation of Fetal Lymphoid Tissue Inducer Cells. Nat. Immunol. 5, 64–73. doi: 10.1038/ni1022 14691482

[B55] Edwards L. A. Nistala K. Mills D. C. Stephenson H. N. Zilbauer M. Wren B. W.. (2010). Delineation of the Innate and Adaptive T-Cell Immune Outcome in the Human Host in Response to Campylobacter Jejuni Infection. PloS One 5, e15398. doi: 10.1371/journal.pone.0015398 21085698 PMC2976761

[B56] Eriguchi Y. Nakamura K. Yokoi Y. Sugimoto R. Takahashi S. Hashimoto D.. (2018). Essential Role of Ifn-γ in T Cell-Associated Intestinal Inflammation. JCI Insight 3 (18), e121886. doi: 10.1172/jci.insight.121886 PMC6237234 30232288

[B57] Feng C. G. Kaviratne M. Rothfuchs A. G. Cheever A. Hieny S. Young H. A.. (2006). Nk Cell-Derived Ifn-Gamma Differentially Regulates Innate Resistance and Neutrophil Response in T Cell-Deficient Hosts Infected With Mycobacterium Tuberculosis. J. Immunol. (Baltimore Md. 1950) 177, 7086–7093. doi: 10.4049/jimmunol.177.10.7086 17082625

[B58] Ferreira A. C. F. Szeto A. C. H. Heycock M. W. D. Clark P. A. Walker J. A. Crisp A.. (2021). Rorα is a Critical Checkpoint for T Cell and Ilc2 Commitment in the Embryonic Thymus. Nat. Immunol. 22, 166–178. doi: 10.1038/s41590-020-00833-w 33432227 PMC7116838

[B59] Fimlaid K. A. Lindow J. C. Tribble D. R. Bunn J. Y. Maue A. C. Kirkpatrick B. D. (2014). Peripheral Cd4+ T Cell Cytokine Responses Following Human Challenge and Re-Challenge With Campylobacter Jejuni. PloS One 9, e112513. doi: 10.1371/journal.pone.0112513 25397604 PMC4232357

[B60] Fonseca D. M. Hand T. W. Han S. J. Gerner M. Y. Glatman Zaretsky A. Byrd A. L.. (2015). Microbiota-Dependent Sequelae of Acute Infection Compromise Tissue-Specific Immunity. Cell 163, 354–366. doi: 10.1016/j.cell.2015.08.030 26451485 PMC4826740

[B61] Forkel M. Mjosberg J. (2016). Dysregulation of Group 3 Innate Lymphoid Cells in the Pathogenesis of Inflammatory Bowel Disease. Curr. Allergy Asthma Rep. 16, 73. doi: 10.1007/s11882-016-0652-3 27645534 PMC5028403

[B62] Forkel M. van Tol S. Hoog C. Michaelsson J. Almer S. Mjosberg J. (2019). Distinct Alterations in the Composition of Mucosal Innate Lymphoid Cells in Newly Diagnosed and Established Crohn's Disease and Ulcerative Colitis. J. Crohns Colitis 13, 67–78. doi: 10.1093/ecco-jcc/jjy119 30496425

[B63] Frucht D. M. Fukao T. Bogdan C. Schindler H. O'Shea J. J. Koyasu S. (2001). Ifn-Gamma Production by Antigen-Presenting Cells: Mechanisms Emerge. Trends Immunol. 22, 556–560. doi: 10.1016/s1471-4906(01)02005-1 11574279

[B64] Fuchs A. Vermi W. Lee J. S. Lonardi S. Gilfillan S. Newberry R. D.. (2013). Intraepithelial Type 1 Innate Lymphoid Cells are a Unique Subset of Il-12- and Il-15-Responsive Ifn-Gamma-Producing Cells. Immunity 38, 769–781. doi: 10.1016/j.immuni.2013.02.010 23453631 PMC3634355

[B65] Gao Y. Souza-Fonseca-Guimaraes F. Bald T. Ng S. S. Young A. Ngiow S. F.. (2017). Tumor Immunoevasion by the Conversion of Effector Nk Cells Into Type 1 Innate Lymphoid Cells. Nat. Immunol. 18, 1004–1015. doi: 10.1038/ni.3800 28759001

[B66] Gasteiger G. Fan X. Dikiy S. Lee S. Y. Rudensky A. Y. (2015). Tissue Residency of Innate Lymphoid Cells in Lymphoid and Nonlymphoid Organs. Science 350, 981–985. doi: 10.1126/science.aac9593 26472762 PMC4720139

[B67] Gazzinelli R. T. Wysocka M. Hayashi S. Denkers E. Y. Hieny S. Caspar P.. (1994). Parasite-Induced Il-12 Stimulates Early Ifn-Gamma Synthesis and Resistance During Acute Infection With Toxoplasma Gondii. J. Immunol. 153(6), 2533–2543. 7915739

[B68] Geremia A. Arancibia-Cárcamo C. V. Fleming M. P. Rust N. Singh B. Mortensen N. J.. (2011). Il-23-Responsive Innate Lymphoid Cells are Increased in Inflammatory Bowel Disease. J. Exp. Med. 208, 1127–1133. doi: 10.1084/jem.20101712 21576383 PMC3173242

[B69] Goldszmid R. S. Bafica A. Jankovic D. Feng C. G. Caspar P. Winkler-Pickett R.. (2007). Tap-1 Indirectly Regulates Cd4+ T Cell Priming in Toxoplasma Gondii Infection by Controlling Nk Cell Ifn-Gamma Production. J. Exp. Med. 204, 2591–2602. doi: 10.1084/jem.20070634 17923502 PMC2118487

[B70] Goldszmid R. S. Caspar P. Rivollier A. White S. Dzutsev A. Hieny S.. (2012). Nk Cell-Derived Interferon-Gamma Orchestrates Cellular Dynamics and the Differentiation of Monocytes Into Dendritic Cells at the Site of Infection. Immunity 36, 1047–1059. doi: 10.1016/j.immuni.2012.03.026 22749354 PMC3412151

[B71] Golebski K. Layhadi J. A. Sahiner U. Steveling-Klein E. H. Lenormand M. M. Li R. C. Y.. (2021). Induction of Il-10-Producing Type 2 Innate Lymphoid Cells by Allergen Immunotherapy is Associated With Clinical Response. Immunity 54, 291–307.e7. doi: 10.1016/j.immuni.2020.12.013 33450188

[B72] Gordon S. M. Chaix J. Rupp L. J. Wu J. Madera S. Sun J. C.. (2012). The Transcription Factors T-Bet and Eomes Control Key Checkpoints of Natural Killer Cell Maturation. Immunity 36, 55–67. doi: 10.1016/j.immuni.2011.11.016 22261438 PMC3381976

[B73] Goto Y. Obata T. Kunisawa J. Sato S. Ivanov I. I. Lamichhane A.. (2014). Innate Lymphoid Cells Regulate Intestinal Epithelial Cell Glycosylation. Science 345:1254009. doi: 10.1126/science.1254009 25214634 PMC4774895

[B74] Gradel K. O. Nielsen H. L. Schonheyder H. C. Ejlertsen T. Kristensen B. Nielsen H. (2009). Increased Short- and Long-Term Risk of Inflammatory Bowel Disease After Salmonella or Campylobacter Gastroenteritis. Gastroenterology 137, 495–501. doi: 10.1053/j.gastro.2009.04.001 19361507

[B75] Halim T. Y. Krauss R. H. Sun A. C. Takei F. (2012). Lung Natural Helper Cells are a Critical Source of Th2 Cell-Type Cytokines in Protease Allergen-Induced Airway Inflammation. Immunity 36, 451–463. doi: 10.1016/j.immuni.2011.12.020 22425247

[B76] Hameed A. (2019). Human Immunity Against Campylobacter Infection. Immune Netw. 19, e38. doi: 10.4110/in.2019.19.e38 31921468 PMC6943174

[B77] Handley S. A. Newberry R. D. Miller V. L. (2005). Yersinia Enterocolitica Invasin-Dependent and Invasin-Independent Mechanisms of Systemic Dissemination. Infect. Immun. 73, 8453–8455. doi: 10.1128/iai.73.12.8453-8455.2005 16299350 PMC1307041

[B78] Heimesaat M. M. Grundmann U. Alutis M. E. Fischer A. Gobel U. B. Bereswill S. (2016). The Il-23/Il-22/Il-18 Axis in Murine Campylobacter Jejuni Infection. Gut Pathog. 8, 21. doi: 10.1186/s13099-016-0106-4 27385977 PMC4934010

[B79] Hepworth M. R. Fung T. C. Masur S. H. Kelsen J. R. McConnell F. M. Dubrot J.. (2015). Immune Tolerance. Group 3 Innate Lymphoid Cells Mediate Intestinal Selection of Commensal Bacteria-Specific Cd4(+) T Cells. Science 348, 1031–1035. doi: 10.1126/science.aaa4812 25908663 PMC4449822

[B80] Hepworth M. R. Monticelli L. A. Fung T. C. Ziegler C. G. Grunberg S. Sinha R.. (2013). Innate Lymphoid Cells Regulate Cd4+ T-Cell Responses to Intestinal Commensal Bacteria. Nature 498, 113–117. doi: 10.1038/nature12240 23698371 PMC3699860

[B81] Hering N. A. Richter J. F. Krug S. M. Günzel D. Fromm A. Bohn E.. (2011). Yersinia Enterocolitica Induces Epithelial Barrier Dysfunction Through Regional Tight Junction Changes in Colonic Ht-29/B6 Cell Monolayers. Lab. Invest. J. Tech. Methods Pathol. 91, 310–324. doi: 10.1038/labinvest.2010.180 20956974

[B82] He Y. Xu H. Song C. Koprivsek J. J. Arulanandam B. Yang H.. (2021). Adoptive Transfer of Group 3-Like Innate Lymphoid Cells Restores Mouse Colon Resistance to Colonization of a Gamma Interferon-Susceptible Chlamydia Muridarum Mutant. Infect. Immun. 89 (2), e00533–00520. doi: 10.1128/IAI.00533-20 33139384 PMC7822149

[B83] Ho J. Bailey M. Zaunders J. Mrad N. Sacks R. Sewell W.. (2015). Group 2 Innate Lymphoid Cells (Ilc2s) are Increased in Chronic Rhinosinusitis With Nasal Polyps or Eosinophilia. Clin. Exp. Allergy J. Br. Soc. Allergy Clin. Immunol. 45, 394–403. doi: 10.1111/cea.12462 25429730

[B84] Howard E. Lewis G. Galle-Treger L. Hurrell B. P. Helou D. G. Shafiei-Jahani P.. (2021). Il-10 Production by Ilc2s Requires Blimp-1 and Cmaf, Modulates Cellular Metabolism, and Ameliorates Airway Hyperreactivity. J. Allergy Clin. Immunol. 147, 1281–95.e5. doi: 10.1016/j.jaci.2020.08.024 32905799

[B85] Howe S. E. Shillova N. Konjufca V. (2019). Dissemination of Chlamydia From the Reproductive Tract to the Gastro-Intestinal Tract Occurs in Stages and Relies on Chlamydia Transport by Host Cells. PloS Pathog. 15, e1008207. doi: 10.1371/journal.ppat.1008207 31790512 PMC6907867

[B86] Huang Y. Guo L. Qiu J. Chen X. Hu-Li J. Siebenlist U.. (2015). Il-25-Responsive, Lineage-Negative Klrg1(Hi) Cells are Multipotential 'Inflammatory' Type 2 Innate Lymphoid Cells. Nat. Immunol. 16, 161–169. doi: 10.1038/ni.3078 25531830 PMC4297567

[B87] Huang J. Lee H. Y. Zhao X. Han J. Su Y. Sun Q.. (2021). Interleukin-17d Regulates Group 3 Innate Lymphoid Cell Function Through its Receptor Cd93. Immunity 54, 673–686.e4. doi: 10.1016/j.immuni.2021.03.018 33852831

[B88] Huang Y. Mao K. Chen X. Sun M. A. Kawabe T. Li W.. (2018). S1p-Dependent Interorgan Trafficking of Group 2 Innate Lymphoid Cells Supports Host Defense. Science 359, 114–119. doi: 10.1126/science.aam5809 29302015 PMC6956613

[B89] Huang Y. Paul W. E. (2016). Inflammatory Group 2 Innate Lymphoid Cells. Int. Immunol. 28, 23–28. doi: 10.1093/intimm/dxv044 26232596 PMC4715228

[B90] Ibiza S. García-Cassani B. Ribeiro H. Carvalho T. Almeida L. Marques R.. (2016). Glial-Cell-Derived Neuroregulators Control Type 3 Innate Lymphoid Cells and Gut Defence. Nature 535, 440–443. doi: 10.1038/nature18644 27409807 PMC4962913

[B91] Igietseme J. U. Portis J. L. Perry L. L. (2001). Inflammation and Clearance of Chlamydia Trachomatis in Enteric and Nonenteric Mucosae. Infect. Immun. 69, 1832–1840. doi: 10.1128/IAI.69.3.1832-1840.2001 11179361 PMC98090

[B92] Imai Y. Yasuda K. Nagai M. Kusakabe M. Kubo M. Nakanishi K.. (2019). Il-33-Induced Atopic Dermatitis-Like Inflammation in Mice is Mediated by Group 2 Innate Lymphoid Cells in Concert With Basophils. J. Invest. Dermatol. 139, 2185–2194.e3. doi: 10.1016/j.jid.2019.04.016 31121178

[B93] Ivanova D. L. Krempels R. Denton S. L. Fettel K. D. Saltz G. M. Rach D.. (2020). Nk Cells Negatively Regulate Cd8 T Cells to Promote Immune Exhaustion and Chronic Toxoplasma Gondii Infection. Front. Cell Infect. Microbiol. 10, 313. doi: 10.3389/fcimb.2020.00313 32733814 PMC7360721

[B94] Ivashkiv L. B. (2018). Ifngamma: Signalling, Epigenetics and Roles in Immunity, Metabolism, Disease and Cancer Immunotherapy. Nat. Rev. Immunol. 18, 545–558. doi: 10.1038/s41577-018-0029-z 29921905 PMC6340644

[B95] Jing X. Korchagina A. A. Shein S. A. Muraoka W. T. Koroleva E. Tumanov A. V. (2020). Il-23 Contributes to Campylobacter Jejuni-Induced Intestinal Pathology via Promoting Il-17 and Ifngamma Responses by Innate Lymphoid Cells. Front. Immunol. 11, 579615. doi: 10.3389/fimmu.2020.579615 33488580 PMC7815532

[B96] Johansson M. Schön K. Ward M. Lycke N. (1997). Genital Tract Infection With Chlamydia Trachomatis Fails to Induce Protective Immunity in Gamma Interferon Receptor-Deficient Mice Despite a Strong Local Immunoglobulin a Response. Infect. Immun. 65, 1032–1044. doi: 10.1128/iai.65.3.1032-1044.1997 9038313 PMC175085

[B97] Kaakoush N. O. Castaño-Rodríguez N. Mitchell H. M. Man S. M. (2015). Global Epidemiology of Campylobacter Infection. Clin. Microbiol. Rev. 28, 687–720. doi: 10.1128/cmr.00006-15 26062576 PMC4462680

[B98] Kak G. Raza M. Tiwari B. K. (2018). Interferon-Gamma (Ifn-γ): Exploring its Implications in Infectious Diseases. Biomol. Concepts 9, 64–79. doi: 10.1515/bmc-2018-0007 29856726

[B99] Kastele V. Mayer J. Lee E. S. Papazian N. Cole J. J. Cerovic V.. (2021). Intestinal-Derived Ilcs Migrating in Lymph Increase Ifngamma Production in Response to Salmonella Typhimurium Infection. Mucosal Immunol. 14, 717–727. doi: 10.1038/s41385-020-00366-3 33414524 PMC8075955

[B100] Kim B. S. Siracusa M. C. Saenz S. A. Noti M. Monticelli L. A. Sonnenberg G. F.. (2013). Tslp Elicits Il-33-Independent Innate Lymphoid Cell Responses to Promote Skin Inflammation. Sci. Trans. Med. 5, 170ra16. doi: 10.1126/scitranslmed.3005374 PMC3637661 23363980

[B101] Kim T. J. Upadhyay V. Kumar V. Lee K. M. Fu Y. X. (2014). Innate Lymphoid Cells Facilitate Nk Cell Development Through a Lymphotoxin-Mediated Stromal Microenvironment. J. Exp. Med. 211, 1421–1431. doi: 10.1084/jem.20131501 24913234 PMC4076579

[B102] Kiss E. A. Diefenbach A. (2012). Role of the Aryl Hydrocarbon Receptor in Controlling Maintenance and Functional Programs of Rorgammat(+) Innate Lymphoid Cells and Intraepithelial Lymphocytes. Front. Immunol. 3, 124. doi: 10.3389/fimmu.2012.00124 22666222 PMC3364460

[B103] Klose C. S. N. Flach M. Mohle L. Rogell L. Hoyler T. Ebert K.. (2014). Differentiation of Type 1 Ilcs From a Common Progenitor to All Helper-Like Innate Lymphoid Cell Lineages. Cell 157, 340–356. doi: 10.1016/j.cell.2014.03.030 24725403

[B104] Klose C. S. Kiss E. A. Schwierzeck V. Ebert K. Hoyler T. d'Hargues Y.. (2013). A T-Bet Gradient Controls the Fate and Function of Ccr6-Rorgammat+ Innate Lymphoid Cells. Nature 494, 261–265. doi: 10.1038/nature11813 23334414

[B105] Klose C. S. N. Mahlakõiv T. Moeller J. B. Rankin L. C. Flamar A. L. Kabata H.. (2017). The Neuropeptide Neuromedin U Stimulates Innate Lymphoid Cells and Type 2 Inflammation. Nature 549, 282–286. doi: 10.1038/nature23676 28869965 PMC6066372

[B106] Konradt C. Ueno N. Christian D. A. Delong J. H. Pritchard G. H. Herz J.. (2016). Endothelial Cells are a Replicative Niche for Entry of Toxoplasma Gondii to the Central Nervous System. Nat. Microbiol. 1, 16001. doi: 10.1038/nmicrobiol.2016.1 27572166 PMC4966557

[B107] Koprivsek J. J. He Y. Song C. Zhang N. Tumanov A. Zhong G. (2020). Evasion of Innate Lymphoid Cell-Regulated Gamma Interferon Responses by Chlamydia Muridarum to Achieve Long-Lasting Colonization in Mouse Colon. Infect. Immun. 88 (3), e00798–00719. doi: 10.1128/IAI.00798-19 PMC7035925 31818961

[B108] Kruglov A. A. Grivennikov S. I. Kuprash D. V. Winsauer C. Prepens S. Seleznik G. M.. (2013). Nonredundant Function of Soluble Ltα3 Produced by Innate Lymphoid Cells in Intestinal Homeostasis. Science 342, 1243–1246. doi: 10.1126/science.1243364 24311691

[B109] Kullberg M. C. Jankovic D. Feng C. G. Hue S. Gorelick P. L. McKenzie B. S.. (2006). Il-23 Plays a Key Role in Helicobacter Hepaticus-Induced T Cell-Dependent Colitis. J. Exp. Med. 203, 2485–2494. doi: 10.1084/jem.20061082 17030948 PMC2118119

[B110] Kupz A. Scott T. A. Belz G. T. Andrews D. M. Greyer M. Lew A. M.. (2013). Contribution of Thy1+ Nk Cells to Protective Ifn-γ Production During Salmonella Typhimurium Infections. Proc. Natl. Acad. Sci. U.S.A. 110, 2252–2257. doi: 10.1073/pnas.1222047110 23345426 PMC3568339

[B111] Kwong B. Rua R. Gao Y. Flickinger J. Jr. Wang Y. Kruhlak M. J.. (2017). T-Bet-Dependent Nkp46(+) Innate Lymphoid Cells Regulate the Onset of T(H)17-Induced Neuroinflammation. Nat. Immunol. 18, 1117–1127. doi: 10.1038/ni.3816 28805812 PMC5605431

[B112] Lei L. Chen J. Hou S. Ding Y. Yang Z. Zeng H.. (2014). Reduced Live Organism Recovery and Lack of Hydrosalpinx in Mice Infected With Plasmid-Free Chlamydia Muridarum. Infect. Immun. 82, 983–992. doi: 10.1128/IAI.01543-13 24343644 PMC3958014

[B113] Leon-Sicairos N. Reyes-Cortes R. Guadron-Llanos A. M. Maduena-Molina J. Leon-Sicairos C. Canizalez-Roman A. (2015). Strategies of Intracellular Pathogens for Obtaining Iron From the Environment. BioMed. Res. Int. 2015, 476534. doi: 10.1155/2015/476534 26120582 PMC4450229

[B114] Lieberman L. A. Cardillo F. Owyang A. M. Rennick D. M. Cua D. J. Kastelein R. A.. (2004). Il-23 Provides a Limited Mechanism of Resistance to Acute Toxoplasmosis in the Absence of Il-12. J. Immunol. 173, 1887–1893. doi: 10.4049/jimmunol.173.3.1887 15265921

[B115] Lim A. I. Menegatti S. Bustamante J. Le Bourhis L. Allez M. Rogge L.. (2016). Il-12 Drives Functional Plasticity of Human Group 2 Innate Lymphoid Cells. J. Exp. Med. 213, 569–583. doi: 10.1084/jem.20151750 26976630 PMC4821648

[B116] Lippert E. Karrasch T. Sun X. Allard B. Herfarth H. H. Threadgill D.. (2009). Gnotobiotic Il-10; Nf-Kappab Mice Develop Rapid and Severe Colitis Following Campylobacter Jejuni Infection. PloS One 4, e7413. doi: 10.1371/journal.pone.0007413 19841748 PMC2760752

[B117] Lopez-Yglesias A. H. Burger E. Camanzo E. Martin A. T. Araujo A. M. Kwok S. F.. (2021). T-Bet-Dependent Ilc1- and Nk Cell-Derived Ifn-Gamma Mediates Cdc1-Dependent Host Resistance Against Toxoplasma Gondii. PloS Pathog. 17, e1008299. doi: 10.1371/journal.ppat.1008299 33465134 PMC7875365

[B118] Lu G. Zhang R. Geng S. Peng L. Jayaraman P. Chen C.. (2015). Myeloid Cell-Derived Inducible Nitric Oxide Synthase Suppresses M1 Macrophage Polarization. Nat. Commun. 6, 6676. doi: 10.1038/ncomms7676 25813085 PMC4389243

[B119] Macho-Fernandez E. Koroleva E. P. Spencer C. M. Tighe M. Torrado E. Cooper A. M.. (2015). Lymphotoxin Beta Receptor Signaling Limits Mucosal Damage Through Driving Il-23 Production by Epithelial Cells. Mucosal Immunol. 8, 403–413. doi: 10.1038/mi.2014.78 25183367 PMC4364000

[B120] Maggi L. Montaini G. Mazzoni A. Rossettini B. Capone M. Rossi M. C.. (2017). Human Circulating Group 2 Innate Lymphoid Cells can Express Cd154 and Promote Ige Production. J. Allergy Clin. Immunol. 139, 964–76.e4. doi: 10.1016/j.jaci.2016.06.032 27576126

[B121] Malik A. Sharma D. St Charles J. Dybas L. A. Mansfield L. S. (2014). Contrasting Immune Responses Mediate Campylobacter Jejuni-Induced Colitis and Autoimmunity. Mucosal Immunol. 7, 802–817. doi: 10.1038/mi.2013.97 24220299 PMC4112758

[B122] Man S. M. (2011). The Clinical Importance of Emerging Campylobacter Species. *Nature Reviews* . Gastroenterol. Hepatol. 8, 669–685. doi: 10.1038/nrgastro.2011.191 22025030

[B123] Mansfield L. S. Bell J. A. Wilson D. L. Murphy A. J. Elsheikha H. M. Rathinam V. A.. (2007). C57bl/6 and Congenic Interleukin-10-Deficient Mice can Serve as Models of Campylobacter Jejuni Colonization and Enteritis. Infect. Immun. 75, 1099–1115. doi: 10.1128/IAI.00833-06 17130251 PMC1828563

[B124] Mansfield L. S. Patterson J. S. Fierro B. R. Murphy A. J. Rathinam V. A. Kopper J. J.. (2008). Genetic Background of Il-10(-/-) Mice Alters Host-Pathogen Interactions With Campylobacter Jejuni and Influences Disease Phenotype. Microb. Pathogen. 45, 241–257. doi: 10.1016/j.micpath.2008.05.010 18586081 PMC4148907

[B125] Marin N. D. Dunlap M. D. Kaushal D. Khader S. A. (2019). Friend or Foe: The Protective and Pathological Roles of Inducible Bronchus-Associated Lymphoid Tissue in Pulmonary Diseases. J. Immunol. (Baltimore Md. 1950) 202, 2519–2526. doi: 10.4049/jimmunol.1801135 PMC6481307 31010841

[B126] Mashayekhi M. Sandau M. M. Dunay I. R. Frickel E. M. Khan A. Goldszmid R. S.. (2011). Cd8α(+) Dendritic Cells are the Critical Source of Interleukin-12 That Controls Acute Infection by Toxoplasma Gondii Tachyzoites. Immunity 35, 249–259. doi: 10.1016/j.immuni.2011.08.008 21867928 PMC3171793

[B127] May G. R. Sutherland L. R. Meddings J. B. (1993). Is Small Intestinal Permeability Really Increased in Relatives of Patients With Crohn's Disease? Gastroenterology 104, 1627–1632. doi: 10.1016/0016-5085(93)90638-s 8500719

[B128] Mazzurana L. Bonfiglio F. Forkel M. D'Amato M. Halfvarson J. Mjösberg J. (2021). Crohn's Disease is Associated With Activation of Circulating Innate Lymphoid Cells. Inflamm. Bowel Dis. 27, 1128–1138. doi: 10.1093/ibd/izaa316 33295628 PMC8205634

[B129] Mazzurana L. Czarnewski P. Jonsson V. Wigge L. Ringnér M. Williams T. C.. (2021). Tissue-Specific Transcriptional Imprinting and Heterogeneity in Human Innate Lymphoid Cells Revealed by Full-Length Single-Cell Rna-Sequencing. Cell Res. 31, 554–568. doi: 10.1038/s41422-020-00445-x 33420427 PMC8089104

[B130] McCarthy N. Giesecke J. (2001). Incidence of Guillain-Barre Syndrome Following Infection With Campylobacter Jejuni. Am. J. Epidemiol. 153, 610–614. doi: 10.1093/aje/153.6.610 11257070

[B131] McKenzie A. N. (2014). ype-2 Innate Lymphoid Cells in Asthma and Allergy. Ann. Am. Thorac. Soc. 11 (Suppl 5), S263–S270. doi: 10.1513/AnnalsATS.201403-097AW 25525730 PMC4298972

[B132] Mebius R. E. Rennert P. Weissman I. L. (1997). Developing Lymph Nodes Collect Cd4+Cd3- Ltbeta+ Cells That can Differentiate to Apc, Nk Cells, and Follicular Cells But Not T or B Cells. Immunity 7, 493–504. doi: 10.1016/s1074-7613(00)80371-4 9354470

[B133] Melo-Gonzalez F. Hepworth M. R. (2017). Functional and Phenotypic Heterogeneity of Group 3 Innate Lymphoid Cells. Immunology 150, 265–275. doi: 10.1111/imm.12697 27935637 PMC5290240

[B134] Mercado M. A. B. Du W. Malaviarachchi P. A. Gann J. I. Li L. X. (2021). Innate Ifn-Gamma is Essential for Systemic Chlamydia Muridarum Control in Mice, While Cd4 T Cell-Dependent Ifn-Gamma Production is Highly Redundant in the Female Reproductive Tract. Infect. Immun. 89(3), e00541–00520. doi: 10.1128/IAI.00541-20 PMC8097277 33257535

[B135] Metzemaekers M. Vanheule V. Janssens R. Struyf S. Proost P. (2017). Overview of the Mechanisms That may Contribute to the non-Redundant Activities of Interferon-Inducible Cxc Chemokine Receptor 3 Ligands. Front. Immunol. 8, 1970. doi: 10.3389/fimmu.2017.01970 29379506 PMC5775283

[B136] Mikami Y. Scarno G. Zitti B. Shih H. Y. Kanno Y. Santoni A.. (2018). Ncr(+) Ilc3 Maintain Larger Stat4 Reservoir *via* T-Bet to Regulate Type 1 Features Upon Il-23 Stimulation in Mice. Eur. J. Immunol. 48, 1174–1180. doi: 10.1002/eji.201847480 29524223 PMC11170443

[B137] Mjosberg J. Bernink J. Golebski K. Karrich J. J. Peters C. P. Blom B.. (2012). The Transcription Factor Gata3 is Essential for the Function of Human Type 2 Innate Lymphoid Cells. Immunity 37, 649–659. doi: 10.1016/j.immuni.2012.08.015 23063330

[B138] Mjösberg J. M. Trifari S. Crellin N. K. Peters C. P. van Drunen C. M. Piet B.. (2011). Human Il-25- and Il-33-Responsive Type 2 Innate Lymphoid Cells are Defined by Expression of Crth2 and Cd161. Nat. Immunol. 12, 1055–1062. doi: 10.1038/ni.2104 21909091

[B139] Mortha A. Chudnovskiy A. Hashimoto D. Bogunovic M. Spencer S. P. Belkaid Y.. (2014). Microbiota-Dependent Crosstalk Between Macrophages and Ilc3 Promotes Intestinal Homeostasis. Science 343, 1249288. doi: 10.1126/science.1249288 24625929 PMC4291125

[B140] Munoz M. Eidenschenk C. Ota N. Wong K. Lohmann U. Kuhl A. A.. (2015). Interleukin-22 Induces Interleukin-18 Expression From Epithelial Cells During Intestinal Infection. Immunity 42, 321–331. doi: 10.1016/j.immuni.2015.01.011 25680273

[B141] Munoz M. Heimesaat M. M. Danker K. Struck D. Lohmann U. Plickert R.. (2009). Interleukin (Il)-23 Mediates Toxoplasma Gondii-Induced Immunopathology in the Gut *via* Matrixmetalloproteinase-2 and Il-22 But Independent of Il-17. J. Exp. Med. 206, 3047–3059. doi: 10.1084/jem.20090900 19995958 PMC2806449

[B142] Munoz M. Liesenfeld O. Heimesaat M. M. (2011). Immunology of Toxoplasma Gondii. Immunol. Rev. 240, 269–285. doi: 10.1111/j.1600-065X.2010.00992.x 21349099

[B143] Muraoka W. T. Korchagina A. A. Xia Q. Shein S. A. Jing X. Lai Z.. (2021). Campylobacter Infection Promotes Ifngamma-Dependent Intestinal Pathology *via* Ilc3 to Ilc1 Conversion. Mucosal Immunol. 14, 703–716. doi: 10.1038/s41385-020-00353-8 33214656 PMC8084871

[B144] Nachamkin I. (2002). Chronic Effects of Campylobacter Infection. Microbes Infect. 4, 399–403. doi: 10.1016/s1286-4579(02)01553-8 11932190

[B145] Nagashima H. Mahlakõiv T. Shih H. Y. Davis F. P. Meylan F. Huang Y.. (2019). Neuropeptide Cgrp Limits Group 2 Innate Lymphoid Cell Responses and Constrains Type 2 Inflammation. Immunity 51, 682–695.e6. doi: 10.1016/j.immuni.2019.06.009 31353223 PMC6801073

[B146] Nava P. Koch S. Laukoetter M. G. Lee W. Y. Kolegraff K. Capaldo C. T.. (2010). Interferon-Gamma Regulates Intestinal Epithelial Homeostasis Through Converging Beta-Catenin Signaling Pathways. Immunity 32, 392–402. doi: 10.1016/j.immuni.2010.03.001 20303298 PMC2859189

[B147] Navarro-Llavat M. Domenech E. Bernal I. Sanchez-Delgado J. Manterola J. M. Garcia-Planella E.. (2009). Prospective, Observational, Cross-Sectional Study of Intestinal Infections Among Acutely Active Inflammatory Bowel Disease Patients. Digestion 80, 25–29. doi: 10.1159/000212076 19439968

[B148] Neill D. R. Wong S. H. Bellosi A. Flynn R. J. Daly M. Langford T. K.. (2010). Nuocytes Represent a New Innate Effector Leukocyte That Mediates Type-2 Immunity. Nature 464, 1367–1370. doi: 10.1038/nature08900 20200518 PMC2862165

[B149] Ngo Thi Phuong N. Palmieri V. Adamczyk A. Klopfleisch R. Langhorst J. Hansen W.. (2021). Il-33 Drives Expansion of Type 2 Innate Lymphoid Cells and Regulatory T Cells and Protects Mice From Severe, Acute Colitis. Front. Immunol. 12:669787. doi: 10.3389/fimmu.2021.669787 34335571 PMC8320374

[B150] Odenwald M. A. Turner J. R. (2017). The Intestinal Epithelial Barrier: A Therapeutic Target? *Nature Reviews* . Gastroenterol. Hepatol. 14, 9–21. doi: 10.1038/nrgastro.2016.169 PMC5554468 27848962

[B151] Okamura H. Kashiwamura S. Tsutsui H. Yoshimoto T. Nakanishi K. (1998). Regulation of Interferon-Gamma Production by Il-12 and Il-18. Curr. Opin. Immunol. 10, 259–264. doi: 10.1016/s0952-7915(98)80163-5 9638361

[B152] Onder L. Ludewig B. (2018). A Fresh View on Lymph Node Organogenesis. Trends Immunol. 39, 775–787. doi: 10.1016/j.it.2018.08.003 30150089

[B153] Pahari S. Khan N. Aqdas M. Negi S. Kaur J. Agrewala J. N. (2016). Infergen Stimulated Macrophages Restrict Mycobacterium Tuberculosis Growth by Autophagy and Release of Nitric Oxide. Sci. Rep. 6, 39492. doi: 10.1038/srep39492 28000752 PMC5175149

[B154] Palm N. W. Rosenstein R. K. Medzhitov R. (2012). Allergic Host Defences. Nature 484, 465–472. doi: 10.1038/nature11047 22538607 PMC3596087

[B155] Pan L. Chen X. Liu X. Qiu W. Liu Y. Jiang W.. (2021). Innate Lymphoid Cells Exhibited Il-17-Expressing Phenotype in Active Tuberculosis Disease. BMC Pulmon. Med. 21, 318. doi: 10.1186/s12890-021-01678-1 PMC8513179 34641843

[B156] Pappu R. Rutz S. Ouyang W. (2012). Regulation of Epithelial Immunity by Il-17 Family Cytokines. Trends Immunol. 33, 343–349. doi: 10.1016/j.it.2012.02.008 22476048

[B157] Park E. Patel S. Wang Q. Andhey P. Zaitsev K. Porter S.. (2019). Toxoplasma Gondii Infection Drives Conversion of Nk Cells Into Ilc1-Like Cells. eLife 8, e47605. doi: 10.7554/eLife.47605 31393266 PMC6703900

[B158] Perry L. L. Feilzer K. Caldwell H. D. (1997). Immunity to Chlamydia Trachomatis is Mediated by T Helper 1 Cells Through Ifn-Gamma-Dependent and -Independent Pathways. J. Immunol. 158 (7), 3344–3352. 9120292

[B159] Pham O. H. McSorley S. J. (2015). Protective Host Immune Responses to Salmonella Infection. Future Microbiol. 10, 101–110. doi: 10.2217/fmb.14.98 25598340 PMC4323267

[B160] Podinovskaia M. Lee W. Caldwell S. Russell D. G. (2013). Infection of Macrophages With Mycobacterium Tuberculosis Induces Global Modifications to Phagosomal Function. Cell. Microbiol. 15, 843–859. doi: 10.1111/cmi.12092 23253353 PMC3620910

[B161] Popov A. Abdullah Z. Wickenhauser C. Saric T. Driesen J. Hanisch F. G.. (2006). Indoleamine 2,3-Dioxygenase-Expressing Dendritic Cells Form Suppurative Granulomas Following Listeria Monocytogenes Infection. J. Clin. Invest. 116, 3160–3170. doi: 10.1172/JCI28996 17111046 PMC1636691

[B162] Price A. E. Liang H. E. Sullivan B. M. Reinhardt R. L. Eisley C. J. Erle D. J.. (2010). Systemically Dispersed Innate Il-13-Expressing Cells in Type 2 Immunity. Proc. Natl. Acad. Sci. U.S.A. 107, 11489–11494. doi: 10.1073/pnas.1003988107 20534524 PMC2895098

[B163] Pu Q. Lin P. Gao P. Wang Z. Guo K. Qin S.. (2021). Gut Microbiota Regulate Gut-Lung Axis Inflammatory Responses by Mediating Ilc2 Compartmental Migration. J. Immunol. doi: 10.4049/jimmunol.2001304 PMC8674377 34135060

[B164] Qiu J. Heller J. J. Guo X. Chen Z. M. Fish K. Fu Y. X.. (2012). The Aryl Hydrocarbon Receptor Regulates Gut Immunity Through Modulation of Innate Lymphoid Cells. Immunity 36, 92–104. doi: 10.1016/j.immuni.2011.11.011 22177117 PMC3268875

[B165] Rank R. G. Yeruva L. (2014). Hidden in Plain Sight: Chlamydial Gastrointestinal Infection and its Relevance to Persistence in Human Genital Infection. Infect. Immun. 82, 1362–1371. doi: 10.1128/iai.01244-13 24421044 PMC3993372

[B166] Rao A. Strauss O. Kokkinou E. Bruchard M. Tripathi K. P. Schlums H.. (2020). Cytokines Regulate the Antigen-Presenting Characteristics of Human Circulating and Tissue-Resident Intestinal Ilcs. Nat. Commun. 11, 2049. doi: 10.1038/s41467-020-15695-x 32341343 PMC7184749

[B167] Reynders A. Yessaad N. Vu Manh T. P. Dalod M. Fenis A. Aubry C.. (2011). Identity, Regulation and In Vivo Function of Gut Nkp46+Rorgammat+ and Nkp46+Rorgammat- Lymphoid Cells. EMBO J. 30, 2934–2947. doi: 10.1038/emboj.2011.201 21685873 PMC3160256

[B168] Robinette M. L. Cella M. Telliez J. B. Ulland T. K. Barrow A. D. Capuder K.. (2018). Jak3 Deficiency Blocks Innate Lymphoid Cell Development. Mucosal Immunol. 11, 50–60. doi: 10.1038/mi.2017.38 28513593 PMC5693788

[B169] Romero-Suárez S. Del Rio Serrato A. Bueno R. J. Brunotte-Strecker D. Stehle C. Figueiredo C. A.. (2019). The Central Nervous System Contains Ilc1s That Differ From Nk Cells in the Response to Inflammation. Front. Immunol. 10, 2337. doi: 10.3389/fimmu.2019.02337 31649664 PMC6795712

[B170] Rottenberg M. E. Gigliotti Rothfuchs A. Gigliotti D. Ceausu M. Une C. Levitsky V.. (2000). Regulation and Role of Ifn-Gamma in the Innate Resistance to Infection With Chlamydia Pneumoniae. J. Immunol. (Baltimore Md. 1950) 164, 4812–4818. doi: 10.4049/jimmunol.164.9.4812 10779789

[B171] Rottenberg M. E. Gigliotti-Rothfuchs A. Wigzell H. (2002). The Role of Ifn-Gamma in the Outcome of Chlamydial Infection. Curr. Opin. Immunol. 14, 444–451. doi: 10.1016/s0952-7915(02)00361-8 12088678

[B172] Salimi M. Barlow J. L. Saunders S. P. Xue L. Gutowska-Owsiak D. Wang X.. (2013). A Role for Il-25 and Il-33-Driven Type-2 Innate Lymphoid Cells in Atopic Dermatitis. J. Exp. Med. 210, 2939–2950. doi: 10.1084/jem.20130351 24323357 PMC3865470

[B173] Sanos S. L. Bui V. L. Mortha A. Oberle K. Heners C. Johner C.. (2009). Rorgammat and Commensal Microflora are Required for the Differentiation of Mucosal Interleukin 22-Producing Nkp46+ Cells. Nat. Immunol. 10, 83–91. doi: 10.1038/ni.1684 19029903 PMC4217274

[B174] Santos R. L. Bäumler A. J. (2004). Cell Tropism of Salmonella Enterica. Int. J. Med. Microbiol. IJMM 294, 225–233. doi: 10.1016/j.ijmm.2004.06.029 15532980

[B175] Satoh-Takayama N. Kato T. Motomura Y. Kageyama T. Taguchi-Atarashi N. Kinoshita-Daitoku R.. (2020). Bacteria-Induced Group 2 Innate Lymphoid Cells in the Stomach Provide Immune Protection Through Induction of Iga. Immunity 52, 635–649.e4. doi: 10.1016/j.immuni.2020.03.002 32240600

[B176] Scandella E. Bolinger B. Lattmann E. Miller S. Favre S. Littman D. R.. (2008). Restoration of Lymphoid Organ Integrity Through the Interaction of Lymphoid Tissue-Inducer Cells With Stroma of the T Cell Zone. Nat. Immunol. 9, 667–675. doi: 10.1038/ni.1605 18425132

[B177] Scharton-Kersten T. M. Wynn T. A. Denkers E. Y. Bala S. Grunvald E. Hieny S.. (1996). In the Absence of Endogenous Ifn-Gamma, Mice Develop Unimpaired Il-12 Responses to Toxoplasma Gondii While Failing to Control Acute Infection. J. Immunol. 157(9), 4045–4054. 8892638

[B178] Schmitz H. Barmeyer C. Fromm M. Runkel N. Foss H. D. Bentzel C. J.. (1999). Altered Tight Junction Structure Contributes to the Impaired Epithelial Barrier Function in Ulcerative Colitis. Gastroenterology 116, 301–309. doi: 10.1016/s0016-5085(99)70126-5 9922310

[B179] Schroder K. Hertzog P. J. Ravasi T. Hume D. A. (2004). Interferon-Gamma: An Overview of Signals, Mechanisms and Functions. J. Leukoc. Biol. 75, 163–189. doi: 10.1189/jlb.0603252 14525967

[B180] Schwartz D. M. Kanno Y. Villarino A. Ward M. Gadina M. O'Shea J. J. (2017). Jak Inhibition as a Therapeutic Strategy for Immune and Inflammatory Diseases. Nat. Rev. Drug Discov 17, 78. doi: 10.1038/nrd.2017.267 PMC6168198 29282366

[B181] Seehus C. R. Kadavallore A. Torre B. Yeckes A. R. Wang Y. Tang J.. (2017). Alternative Activation Generates Il-10 Producing Type 2 Innate Lymphoid Cells. Nat. Commun. 8, 1900. doi: 10.1038/s41467-017-02023-z 29196657 PMC5711851

[B182] Seillet C. Luong K. Tellier J. Jacquelot N. Shen R. D. Hickey P.. (2020). The Neuropeptide Vip Confers Anticipatory Mucosal Immunity by Regulating Ilc3 Activity. Nat. Immunol. 21, 168–177. doi: 10.1038/s41590-019-0567-y 31873294

[B183] Seo G. Y. Giles D. A. Kronenberg M. (2020). The Role of Innate Lymphoid Cells in Response to Microbes at Mucosal Surfaces. Mucosal Immunol. 13, 399–412. doi: 10.1038/s41385-020-0265-y 32047273 PMC7186215

[B184] Seo G. Y. Shui J. W. Takahashi D. Song C. Wang Q. Kim K.. (2018). Light-Hvem Signaling in Innate Lymphoid Cell Subsets Protects Against Enteric Bacterial Infection. Cell Host Microbe 24, 249–60.e4. doi: 10.1016/j.chom.2018.07.008 30092201 PMC6132068

[B185] Shou Y. Koroleva E. Spencer C. M. Shein S. A. Korchagina A. A. Yusoof K. A.. (2021). Redefining the Role of Lymphotoxin Beta Receptor in the Maintenance of Lymphoid Organs and Immune Cell Homeostasis in Adulthood. Front. Immunol. 12, 712632. doi: 10.3389/fimmu.2021.712632 34335629 PMC8320848

[B186] Shtrichman R. Samuel C. E. (2001). The Role of Gamma Interferon in Antimicrobial Immunity. Curr. Opin. Microbiol. 4, 251–259. doi: 10.1016/s1369-5274(00)00199-5 11378475

[B187] Silver J. S. Kearley J. Copenhaver A. M. Sanden C. Mori M. Yu L.. (2016). Inflammatory Triggers Associated With Exacerbations of Copd Orchestrate Plasticity of Group 2 Innate Lymphoid Cells in the Lungs. Nat. Immunol. 17, 626–635. doi: 10.1038/ni.3443 27111143 PMC5345745

[B188] Simic M. Manosalva I. Spinelli L. Gentek R. Shayan R. R. Siret C.. (2020). Distinct Waves From the Hemogenic Endothelium Give Rise to Layered Lymphoid Tissue Inducer Cell Ontogeny. Cell Rep. 32, 108004. doi: 10.1016/j.celrep.2020.108004 32783932

[B189] Songhet P. Barthel M. Stecher B. Muller A. J. Kremer M. Hansson G. C.. (2011). Stromal Ifn-Gammar-Signaling Modulates Goblet Cell Function During Salmonella Typhimurium Infection. PloS One 6, e22459. doi: 10.1371/journal.pone.0022459 21829463 PMC3145644

[B190] Stabile H. Scarno G. Fionda C. Gismondi A. Santoni A. Gadina M.. (2018). Jak/stat Signaling in Regulation of Innate Lymphoid Cells: The Gods Before the Guardians. Immunol. Rev. 286, 148–159. doi: 10.1111/imr.12705 30294965 PMC6178832

[B191] Steigler P. Daniels N. J. McCulloch T. R. Ryder B. M. Sandford S. K. Kirman J. R. (2018). Bcg Vaccination Drives Accumulation and Effector Function of Innate Lymphoid Cells in Murine Lungs. Immunol. Cell Biol. 96, 379–389. doi: 10.1111/imcb.12007 29363172

[B192] Steimle V. Siegrist C.-A. Mottet A. Lisowska-Grospierre B. Mach B. (1994). Regulation of Mhc Class Ii Expression by Interferon-γ Mediated by the Transactivator Gene Ciita. Science 265, 106–109. doi: 10.1126/science.8016643 8016643

[B193] Sun X. Threadgill D. Jobin C. (2012). Campylobacter Jejuni Induces Colitis Through Activation of Mammalian Target of Rapamycin Signaling. Gastroenterology 142, 86–95.e5. doi: 10.1053/j.gastro.2011.09.042 21963787 PMC3253301

[B194] Suzuki Y. Orellana M. A. Schreiber R. D. Remington J. S. (1988). Interferon-Gamma: The Major Mediator of Resistance Against Toxoplasma Gondii. Science 240, 516–518. doi: 10.1126/science.3128869 3128869

[B195] Tahoun A. Mahajan S. Paxton E. Malterer G. Donaldson D. S. Wang D.. (2012). Salmonella Transforms Follicle-Associated Epithelial Cells Into M Cells to Promote Intestinal Invasion. Cell Host Microbe 12, 645–656. doi: 10.1016/j.chom.2012.10.009 23159054

[B196] Tait Wojno E. D. Hunter C. A. Stumhofer J. S. (2019). The Immunobiology of the Interleukin-12 Family: Room for Discovery. Immunity 50, 851–870. doi: 10.1016/j.immuni.2019.03.011 30995503 PMC6472917

[B197] Takayama T. Kamada N. Chinen H. Okamoto S. Kitazume M. T. Chang J.. (2010). Imbalance of Nkp44(+)Nkp46(-) and Nkp44(-)Nkp46(+) Natural Killer Cells in the Intestinal Mucosa of Patients With Crohn's Disease. Gastroenterology 139, 882–92, 92.e1-3. doi: 10.1053/j.gastro.2010.05.040 20638936

[B198] Talbot J. Hahn P. Kroehling L. Nguyen H. Li D. Littman D. R. (2020). Feeding-Dependent Vip Neuron-Ilc3 Circuit Regulates the Intestinal Barrier. Nature 579, 575–580. doi: 10.1038/s41586-020-2039-9 32050257 PMC7135938

[B199] Tang L. Zhang H. Lei L. Gong S. Zhou Z. Baseman J.. (2013). Oviduct Infection and Hydrosalpinx in Dba1/J Mice is Induced by Intracervical But Not Intravaginal Inoculation With Chlamydia Muridarum. PloS One 8, e71649. doi: 10.1371/journal.pone.0071649 23940777 PMC3734308

[B200] Thakur A. Mikkelsen H. Jungersen G. (2019). Intracellular Pathogens: Host Immunity and Microbial Persistence Strategies. J. Immunol. Res. 2019:1356540. doi: 10.1155/2019/1356540 31111075 PMC6487120

[B201] Tian Q. Zhou Z. Wang L. Abu-Khdeir A. H. Huo Z. Sun X.. (2020). Gastrointestinal Coinfection Promotes Chlamydial Pathogenicity in the Genital Tract. Infect. Immun. 88 (4), e00905–00919. doi: 10.1128/iai.00905-19 PMC7093119 31988173

[B202] Tominaga K. Yoshimoto T. Torigoe K. Kurimoto M. Matsui K. Hada T.. (2000). Il-12 Synergizes With Il-18 or Il-1β for Ifn-γ Production From Human T Cells. Int. Immunol. 12, 151–160. doi: 10.1093/intimm/12.2.151 10653850

[B203] Tribble D. R. Baqar S. Scott D. A. Oplinger M. L. Trespalacios F. Rollins D.. (2010). Assessment of the Duration of Protection in Campylobacter Jejuni Experimental Infection in Humans. Infect. Immun. 78, 1750–1759. doi: 10.1128/iai.01021-09 20086085 PMC2849408

[B204] Tseng C. T. Rank R. G. (1998). Role of Nk Cells in Early Host Response to Chlamydial Genital Infection. Infect. Immun. 66, 5867–5875. doi: 10.1128/iai.66.12.5867-5875.1998 9826367 PMC108743

[B205] Tumanov A. V. Koroleva E. P. Guo X. Wang Y. Kruglov A. Nedospasov S.. (2011). Lymphotoxin Controls the Il-22 Protection Pathway in Gut Innate Lymphoid Cells During Mucosal Pathogen Challenge. Cell Host Microbe 10, 44–53. doi: 10.1016/j.chom.2011.06.002 21767811 PMC3375029

[B206] Ulrichs T. Kosmiadi G. A. Trusov V. Jorg S. Pradl L. Titukhina M.. (2004). Human Tuberculous Granulomas Induce Peripheral Lymphoid Follicle-Like Structures to Orchestrate Local Host Defence in the Lung. J. Pathol. 204, 217–228. doi: 10.1002/path.1628 15376257

[B207] van de Pavert S. A. (2021). Lymphoid Tissue Inducer (Lti) Cell Ontogeny and Functioning in Embryo and Adult. Biomed. J. 44, 123–132. doi: 10.1016/j.bj.2020.12.003 33849806 PMC8178546

[B208] van der Gracht E. Zahner S. Kronenberg M. (2016). When Insult is Added to Injury: Cross Talk Between Ilcs and Intestinal Epithelium in Ibd. Mediators Inflamm. 2016, 9765238. doi: 10.1155/2016/9765238 27578924 PMC4989064

[B209] Vasilevsky S. Greub G. Nardelli-Haefliger D. Baud D. (2014). Genital Chlamydia Trachomatis: Understanding the Roles of Innate and Adaptive Immunity in Vaccine Research. Clin. Microbiol. Rev. 27, 346–370. doi: 10.1128/CMR.00105-13 24696438 PMC3993100

[B210] Victor A. R. Nalin A. P. Dong W. McClory S. Wei M. Mao C.. (2017). Il-18 Drives Ilc3 Proliferation and Promotes Il-22 Production *via* Nf-κb. J. Immunol. (Baltimore Md. 1950) 199, 2333–2342. doi: 10.4049/jimmunol.1601554 PMC5624342 28842466

[B211] Villanova F. Flutter B. Tosi I. Grys K. Sreeneebus H. Perera G. K.. (2014). Characterization of Innate Lymphoid Cells in Human Skin and Blood Demonstrates Increase of Nkp44+ Ilc3 in Psoriasis. J. Invest. Dermatol. 134, 984–991. doi: 10.1038/jid.2013.477 24352038 PMC3961476

[B212] Vivier E. Artis D. Colonna M. Diefenbach A. Di Santo J. P. Eberl G.. (2018). Innate Lymphoid Cells: 10 Years on. Cell 174, 1054–1066. doi: 10.1016/j.cell.2018.07.017 30142344

[B213] Vivier E. Spits H. Cupedo T. (2009). Interleukin-22-Producing Innate Immune Cells: New Players in Mucosal Immunity and Tissue Repair? Nat. Rev. Immunol. 9, 229–234. doi: 10.1038/nri2522 19319141

[B214] Vonarbourg C. Mortha A. Bui V. L. Hernandez P. P. Kiss E. A. Hoyler T.. (2010). Regulated Expression of Nuclear Receptor Rorgammat Confers Distinct Functional Fates to Nk Cell Receptor-Expressing Rorgammat(+) Innate Lymphocytes. Immunity 33, 736–751. doi: 10.1016/j.immuni.2010.10.017 21093318 PMC3042726

[B215] Vossenkamper A. Struck D. Alvarado-Esquivel C. Went T. Takeda K. Akira S.. (2004). Both Il-12 and Il-18 Contribute to Small Intestinal Th1-Type Immunopathology Following Oral Infection With Toxoplasma Gondii, But Il-12 is Dominant Over Il-18 in Parasite Control. Eur. J. Immunol. 34, 3197–3207. doi: 10.1002/eji.200424993 15368276

[B216] Walker J. A. McKenzie A. N. (2013). Development and Function of Group 2 Innate Lymphoid Cells. Curr. Opin. Immunol. 25, 148–155. doi: 10.1016/j.coi.2013.02.010 23562755 PMC3776222

[B217] Wallrapp A. Riesenfeld S. J. Burkett P. R. Abdulnour R. E. Nyman J. Dionne D.. (2017). The Neuropeptide Nmu Amplifies Ilc2-Driven Allergic Lung Inflammation. Nature 549, 351–356. doi: 10.1038/nature24029 28902842 PMC5746044

[B218] Wang X. Cai J. Lin B. Ma M. Tao Y. Zhou Y.. (2021). Gpr34-Mediated Sensing of Lysophosphatidylserine Released by Apoptotic Neutrophils Activates Type 3 Innate Lymphoid Cells to Mediate Tissue Repair. Immunity 54, 1123–1136.e8. doi: 10.1016/j.immuni.2021.05.007 34107271

[B219] Wang Y. Koroleva E. P. Kruglov A. A. Kuprash D. V. Nedospasov S. A. Fu Y. X.. (2010). Lymphotoxin Beta Receptor Signaling in Intestinal Epithelial Cells Orchestrates Innate Immune Responses Against Mucosal Bacterial Infection. Immunity 32, 403–413. doi: 10.1016/j.immuni.2010.02.011 20226692 PMC2878123

[B220] Wang L. Zhu C. Zhang T. Tian Q. Zhang N. Morrison S.. (2018). Nonpathogenic Colonization With Chlamydia in the Gastrointestinal Tract as Oral Vaccination for Inducing Transmucosal Protection. Infect. Immun. 86(2), e00630–00617. doi: 10.1128/iai.00630-17 PMC5778366 29133348

[B221] Ward N. L. Umetsu D. T. (2014). A New Player on the Psoriasis Block: Il-17a- and Il-22-Producing Innate Lymphoid Cells. J. Invest. Dermatol. 134, 2305–2307. doi: 10.1038/jid.2014.216 25120146 PMC4134095

[B222] Wassenaar T. M. Engelskirchen M. Park S. Lastovica A. (1997). Differential Uptake and Killing Potential of Campylobacter Jejuni by Human Peripheral Monocytes/Macrophages. Med. Microbiol. Immunol. 186, 139–144. doi: 10.1007/s004300050056 9403842

[B223] Wilson M. S. Feng C. G. Barber D. L. Yarovinsky F. Cheever A. W. Sher A.. (2010). Redundant and Pathogenic Roles for Il-22 in Mycobacterial, Protozoan, and Helminth Infections. J. Immunol. (Baltimore Md. 1950) 184, 4378–4390. doi: 10.4049/jimmunol.0903416 PMC3170015 20220096

[B224] Wong S. H. Walker J. A. Jolin H. E. Drynan L. F. Hams E. Camelo A.. (2012). Transcription Factor Rorα is Critical for Nuocyte Development. Nat. Immunol. 13, 229–236. doi: 10.1038/ni.2208 22267218 PMC3343633

[B225] Wroblewska J. A. Zhang Y. Tang H. Guo X. Nagler C. Fu Y. X. (2017). Cutting Edge: Lymphotoxin Signaling is Essential for Clearance of Salmonella From the Gut Lumen and Generation of Anti-Salmonella Protective Immunity. J. Immunol. (Baltimore Md. 1950) 198, 55–60. doi: 10.4049/jimmunol.1600867 PMC5173428 27913631

[B226] Xaus J. Comalada M. Barrachina M. Herrero C. Goñalons E. Soler C.. (2000). The Expression of Mhc Class Ii Genes in Macrophages Is Cell Cycle Dependent. J. Immunol. 165, 6364–6371. doi: 10.4049/jimmunol.165.11.6364 11086074

[B227] Xiao Q. He J. Lei A. Xu H. Zhang L. Zhou P.. (2021). Ppargamma Enhances Ilc2 Function During Allergic Airway Inflammation via Transcription Regulation of St2. Mucosal Immunol. 14, 468–478. doi: 10.1038/s41385-020-00339-6 32811992

[B228] Xu H. Su X. Zhao Y. Tang L. Chen J. Zhong G. (2020). Innate Lymphoid Cells Are Required for Endometrial Resistance to Chlamydia Trachomatis Infection. Infect. Immun. 88(7), e00152–00120. doi: 10.1128/IAI.00152-20 PMC7309611 32341118

[B229] Yadav J. Dikshit N. Ismaeel S. Qadri A. (2020). Innate Activation of Ifn-γ-Inos Axis During Infection With Salmonella Represses the Ability of T Cells to Produce Il-2. Front. Immunol. 11, 514. doi: 10.3389/fimmu.2020.00514 32269573 PMC7109407

[B230] Yarovinsky F. (2014). Innate Immunity to Toxoplasma Gondii Infection. Nat. Rev. Immunol. 14, 109–121. doi: 10.1038/nri3598 24457485

[B231] Yeruva L. Spencer N. Bowlin A. K. Wang Y. Rank R. G. (2013). Chlamydial Infection of the Gastrointestinal Tract: A Reservoir for Persistent Infection. Pathog. Dis. 68, 88–95. doi: 10.1111/2049-632X.12052 23843274 PMC3751173

[B232] Yin S. Yu J. Hu B. Lu C. Liu X. Gao X.. (2018). Runx3 Mediates Resistance to Intracellular Bacterial Infection by Promoting Il12 Signaling in Group 1 Ilc and Ncr+Ilc3. Front. Immunol. 9, 2101. doi: 10.3389/fimmu.2018.02101 30258450 PMC6144956

[B233] Young K. T. Davis L. M. Dirita V. J. (2007). Campylobacter Jejuni: Molecular Biology and Pathogenesis. Nat. Rev. Microbiol. 5, 665–679. doi: 10.1038/nrmicro1718 17703225

[B234] You Y. Zhang X. Wang X. Yue D. Meng F. Zhu J.. (2020). Ilc2 Proliferated by Il-33 Stimulation Alleviates Acute Colitis in Rag1(-/-) Mouse Through Promoting M2 Macrophage Polarization. J. Immunol. Res. 2020, 5018975. doi: 10.1155/2020/5018975 32676507 PMC7334786

[B235] Yu Q. N. Tan W. P. Fan X. L. Guo Y. B. Qin Z. L. Li C. L.. (2018). Increased Group 2 Innate Lymphoid Cells Are Correlated With Eosinophilic Granulocytes in Patients With Allergic Airway Inflammation. Int. Arch. Allergy Immunol. 176, 124–132. doi: 10.1159/000488050 29642055

[B236] Zarepour M. Bhullar K. Montero M. Ma C. Huang T. Velcich A.. (2013). The Mucin Muc2 Limits Pathogen Burdens and Epithelial Barrier Dysfunction During Salmonella Enterica Serovar Typhimurium Colitis. Infect. Immun. 81, 3672–3683. doi: 10.1128/IAI.00854-13 23876803 PMC3811786

[B237] Zeissig S. Bürgel N. Günzel D. Richter J. Mankertz J. Wahnschaffe U.. (2007). Changes in Expression and Distribution of Claudin 2, 5 and 8 Lead to Discontinuous Tight Junctions and Barrier Dysfunction in Active Crohn's Disease. Gut 56, 61–72. doi: 10.1136/gut.2006.094375 16822808 PMC1856677

[B238] Zeng B. Xing R. Dong C. Xing F. (2020). Commentary: Group 3 Innate Lymphoid Cells Mediate Early Protective Immunity Against Tuberculosis. Front. Immunol. 11, 1925. doi: 10.3389/fimmu.2020.01925 32973795 PMC7482547

[B239] Zhong G. (2021). Chlamydia Overcomes Multiple Gastrointestinal Barriers to Achieve Long-Lasting Colonization. Trends Microbiol. 29, 1004–1012. doi: 10.1016/j.tim.2021.03.011 33865675 PMC8510992

[B240] Zortel T. Schmitt-Graeff A. Kirschnek S. Häcker G. (2018). Apoptosis Modulation in the Immune System Reveals a Role of Neutrophils in Tissue Damage in a Murine Model of Chlamydial Genital Infection. J. Infect. Dis. 217, 1832–1843. doi: 10.1093/infdis/jiy126 29522221

